# Cardiometabolic health and risk of dementia and brain atrophy: a community-based prospective cohort study of 0.5 million adults in China

**DOI:** 10.1016/j.lanwpc.2025.101743

**Published:** 2025-11-18

**Authors:** Clara Bueno Lopez, Andri Iona, Daniel Avery, Iain Turnbull, Ling Yang, Huaidong Du, Yiping Chen, Ningmei Zhang, Junshi Chen, Pei Pei, Jun Lv, Canquing Yu, Dianjianyi Sun, Liming Li, Derrick Bennett, Cornelia van Dujin, Robert Clarke, Zhengming Chen, Fiona Bragg

**Affiliations:** a Clinical Trial Service Unit & Epidemiological Studies Unit, Nuffield Department of Population Health, University of Oxford, Oxford, UK; b NCDs Prevention and Control Department, Sichuan CDC, Sichuan, China; c China National Center for Food Safety Risk Assessment, Chaoyang District, Beijing, China; d Peking University Center for Public Health and Epidemic Preparedness & Response, Beijing, China; e Department of Epidemiology and Biostatistics, School of Public Health, Peking University Health Science Center, Beijing, China; f Key Laboratory of Epidemiology of Major Diseases (Peking University), Ministry of Education, China; g Health Data Research UK Oxford, University of Oxford, Oxford, UK

**Keywords:** Dementia, Brain atrophy, Cardiometabolic health, China, Cohort study

## Abstract

**Background:**

Cardiometabolic health has been associated with dementia risk but prospective evidence is limited in China where the burden of cardiometabolic disease and dementia are rising. We investigate the relevance of markers of cardiometabolic health for risk of dementia and brain atrophy.

**Methods:**

China Kadoorie Biobank is a prospective cohort study involving 512,724 adults aged 30–79 years, recruited in 2004–2008 from 10 diverse regions. During ∼12 years’ follow-up, 1099 dementia and 1418 brain atrophy cases were recorded through linked death registries and health insurance databases. Cox regression yield adjusted hazard ratios (HRs) for incident dementia and brain atrophy associated with markers of cardiometabolic health.

**Findings:**

The incidence rate for dementia and brain atrophy was 19.0 and 25.4 per 100,000 person-years, respectively, higher at older ages, and among males. Prior hypertension (adjusted HR 1.21 [95% CI 1.05–1.39]), diabetes (1.36 [1.13–1.65]) and stroke (2.52 [2.14–2.96]) were associated with higher risks of dementia and brain atrophy (1.30 [1.15–1.47], 1.32 [1.12–1.55], 2.43 [2.12–2.78], respectively). Prior IHD was associated with brain atrophy (1.69 [1.47–1.95]), but not dementia (1.17 [0.97–1.41]).

**Interpretation:**

These findings provide evidence of the relevance of markers of cardiometabolic health for dementia among adults in China, highlighting the importance of preventative strategies for cardiometabolic diseases that may lead to benefits for brain health.

**Funding:**

10.13039/100010269Wellcome Trust, 10.13039/501100000265MRC, 10.13039/501100000274BHF, CR-UK, 10.13039/501100017647Kadoorie Charitable Foundation, Chinese MoST and NSFC.

**Research in context**

**Evidence before this study**

We searched PubMed for articles published in English before October 2024, using the terms (“stroke” OR “ihd” OR “chd” OR “ische∗ heart disease” OR “hypertension” OR “diabetes” OR “systolic blood pressure”) AND (“dementia” OR “brain atrophy”) AND (“china” OR “chinese” OR “east asia∗”) AND (“prospective” OR “longitudinal” OR “cohort”). Reference lists of relevant articles were reviewed. Five large East Asian population studies, relying exclusively on health registry data, were identified, but none reporting on associations in mainland China. All studies found positive associations between markers of cardiometabolic health and risk of dementia and measures of brain atrophy (e.g. brain volume), with mixed evidence for the association between hypertension and dementia, particularly Alzheimer's dementia. No study was found describing associations with clinically reported brain atrophy.

**Added value of this study**

This is the first large-scale prospective cohort study in mainland China to reliably quantify the associations between markers of cardiometabolic health and incident dementia (and its main subtypes) and, to the best of our knowledge, the first in any population to explore associations with clinically reported brain atrophy. Prior hypertension, diabetes, and stroke were positively associated with risk of vascular dementia and brain atrophy in this large Chinese population. Usual systolic blood pressure likewise showed a positive log–linear association with risk of dementia and brain atrophy. Stroke emerged as the strongest risk factor for dementia, associated with a more than doubling of risk and independently associated with higher risk of all dementia subtypes, including a substantially stronger association with Alzheimer's dementia than previous reports. In contrast, there was no clear association of hypertension, diabetes or IHD with non-vascular dementias. The observed associations persisted after controlling for the potential influence of diagnostic bias, confounding and reverse causality.

**Implications of all the available evidence**

Markers of cardiometabolic health are important risk factors for dementia in the large, aging—yet relatively understudied—Chinese population, highlighting the potential value for brain health of preventative strategies targeting cardiometabolic diseases. The high burden of these risk factors in China, and in particular of vascular disease and stroke, highlights the added importance of controlling intermediate risk factors, such as blood pressure, for prevention of stroke and maintaining brain health.

## Introduction

Worldwide, the number of people living with dementia is growing rapidly, driven in part by increased life expectancy.^1^, ^2^, ^3^ China faces one of the largest dementia-associated burdens, accounting for approximately one-quarter of global cases.^2^ Furthermore, in contrast with declining age-standardised dementia incidence rates in many Western countries, incidence rates in China have increased by an estimated 25% since 1990.^4^ Despite this substantial and increasing burden, research on the distribution and risk factors of dementia in China remains limited.

Dementia is an umbrella term for a group of symptoms caused by various neurodegenerative diseases, each with distinct aetiologies and potentially differing risk factors. Preventative efforts for dementia focus largely on reducing a growing number of postulated modifiable risk factors, identified through observational epidemiological research. Among the most widely studied are markers of cardiometabolic health, including diabetes, blood pressure, and pre-existing cardiovascular diseases.^5^, ^6^, ^7^ These are postulated to influence dementia risk through various pathways, including chronic inflammation, altered cerebral blood flow and brain metabolism, and predisposition to cerebral small vessel disease and amyloid angiopathy.^5^^,^^8^, ^9^, ^10^ However, while the link between these factors and vascular dementia (VD) is well-established,^10^, ^11^, ^12^ their relationships with other dementia subtypes or brain atrophy (as a proxy of brain health) are less clear, particularly in East Asian populations.^7^^,^^13^^,^^14^ Moreover, boundaries between dementia subtypes often overlap, and multiple subtypes can co-exist. In recent years, the increased use of brain imaging in clinical settings has led to more frequent identification of brain atrophy, defined as decreased brain volume unrelated to focal injury, trauma or infarction,^15^^,^^16^ but its clinical relevance and determinants remain inadequately understood. To date, no large-scale prospective epidemiological studies have explored the relationship between cardiometabolic health and clinically reported brain atrophy. Despite the worldwide relevance of dementia, most studies investigating its risk factors have been in high-income Western countries, with unclear generalisability to populations elsewhere, including China where risk exposures (e.g. high sodium diet,^17^ genetic architecture,^18^ access to healthcare, diagnosis, and treatment^19^^,^^20^) and disease rates (e.g., high stroke incidence rates^6^) differ importantly from those in many Western populations, underscoring the need for evidence from diverse populations.

The present study aims to address these evidence gaps using data from the China Kadoorie Biobank (CKB) prospective cohort study of disability-free, community-dwelling adults to: (i) examine the distribution of dementia, its main subtypes, and brain atrophy; and (ii) investigate the association of markers of cardiometabolic health with dementia and clinically diagnosed brain atrophy.

## Methods

### Study population

Details of the CKB study design and participants have been reported previously.^21^^,^^22^ Briefly, 512,724 adults (aged 30–79) were recruited between 2004 and 2008 from 10 diverse regions (five urban and five rural) across China. In each region, all eligible participants without disability were invited to participate. Prior international, national and local ethics approval was obtained, and all participants provided informed written consent.

### Data collection

At the baseline survey, trained health workers administered laptop-based questionnaires collecting information on sociodemographic characteristics (ethnicity data were not collected), lifestyle (e.g., smoking, alcohol consumption, diet, and physical activity), and personal and family medical history. Trained technicians, using standard protocols and calibrated instruments, recorded a range of physical measurements (e.g., height, weight and blood pressure). Blood pressure was measured twice using a UA–779 digital sphygmomanometer after sitting rested for 5 min. If the difference between the two measurements was >10 mmHg, a third measurement was taken, and the mean of the last two readings was used. Non-fasting blood samples were collected for long-term storage and immediate on-site random plasma glucose (RPG) testing using regularly calibrated Johnson & Johnson SureStep Plus meters (Lifescan, Milpitas, CA, USA). Irrespective of self-reported prior doctor-diagnosed diabetes status, individuals with an RPG level ≥7.8 and < 11.1 mmol/L were invited back the following day for a fasting plasma glucose (FPG) test.

Resurveys, undertaken in 2008 and 2013–2014 among a randomly-selected ∼5% sample of surviving participants (∼80% response rate), collected the same data as at baseline.

### Markers of cardiometabolic health

At recruitment, participants reported on doctor diagnoses of hypertension, diabetes, ischaemic heart disease (IHD), and stroke/transient ischaemic attack (TIA). Among participants without self-reported hypertension, screen-detected hypertension was defined as systolic blood pressure (SBP) ≥140 mmHg or diastolic blood pressure (DBP) ≥90 mmHg. All participants, regardless of self-reported hypertension diagnosis, were asked about recent use (within the last two days) of blood pressure-lowering medication, and an affirmative answer was used to define treated hypertension. Among those without self-reported diabetes, screen-detected diabetes was defined as (1) RPG ≥11.1 mmol/L with <8 h since last eating; or (2) RPG ≥7.0 mmol/L with ≥8 h since last eating; or (3) FPG ≥7.0 mmol/L on subsequent testing for participants with an RPG level between ≥7.8 and < 11.1 mmol/L. Participants with self-reported diabetes were asked about current use of glucose-lowering medications (insulin, metformin or chlorpropamide).

In this report, ‘baseline’ cardiometabolic exposures refers to prevalent conditions at recruitment defined by self-reported previous diagnosis for IHD and stroke/TIA, and self-reported or screen-detected diagnoses for diabetes and hypertension. ‘Incident’ hypertension, diabetes, IHD and stroke occurring >1 year prior to dementia or brain atrophy diagnosis were defined using ICD-10 codes obtained from health insurance data or disease registries among participants without the respective exposure at recruitment (eTable 1). ‘Prior’ IHD, stroke/TIA, hypertension and diabetes included both conditions identified at ‘baseline’ and ‘incident’ diagnoses occurring >1 year prior to dementia or brain atrophy diagnosis.

### Follow-up for dementias and brain atrophy

All participants are followed-up through linkage, via unique national identification number, to disease (for stroke, IHD, cancer and diabetes) and death registries, as well as health insurance databases which covered 98% of study participants. Underlying cause of death is extracted from death certificates. Linkage to the health insurance system provides information on diagnoses resulting in, or during, hospital admissions. Participants not covered by the health insurance system (2%) were followed-up actively via telephone and home visits collecting information on hospitalisation and major health outcomes, ensuring completeness of follow-up for these participants. Clinical adjudication was undertaken systematically for several major diseases, including any hospital-reported cases of first stroke. Medical records, including brain imaging reports, were retrieved and reviewed by specialists, and comorbidities ascertained from the adjudication process were incorporated into follow-up data.^20^

All-cause dementia (ACD), an umbrella term used to refer to VD, Alzheimer's dementia (AD), other/unspecified dementia, and brain atrophy, was identified through ICD-10 codes recorded in health insurance databases or on death certificates (eTable 1).

### Statistical analyses

All 512,724 CKB participants were included in analyses describing the distribution of dementia and brain atrophy. Directly standardised incidence rates and 95% confidence intervals (CI) were calculated based on gamma approximation,^23^ by baseline age, sex, region, and education level. Rates were standardised by age-at-risk (5-year groups), sex and region (urban or rural), as appropriate.

For prospective analyses, participants with missing body mass index (BMI) data (n = 2) were excluded, leaving 512,722 participants. Prevalence and mean values of baseline characteristics were calculated separately among males and females, standardised by age (5-year groups) and the 10 regions, as appropriate.

Adjusted HRs for the associations between prior cardiometabolic exposures (prevalent conditions at recruitment or incident diagnoses recorded in health insurance data or disease registries among participants without the respective exposure at recruitment) and first occurrence of dementia or brain atrophy were estimated using time-dependent Cox proportional hazard models,^24^^,^^25^ using age at first incidence to determine the time range between exposure and outcome, and censoring participants who developed the outcome within one year of the exposure. HRs were stratified by age-at-risk (5-year groups), sex, and region, and adjusted for smoking (never/ever regular), alcohol drinking (never/ever regular), highest level of education (no formal school, primary school, middle school, high school, technical school/college, university), physical activity (metabolic equivalent of task [MET] hours per day), BMI (continuous scale), and a healthy diet score (ranging from 0 to 9).^26^, ^27^, ^28^, ^29^, ^30^ All covariates were selected a priori based on evidence from published literature of their associations with markers of cardiometabolic health and with dementia.^5^^,^^6^^,^^8^^,^^31^^,^^32^ Analyses were restricted to first events occurring at ages 40–89 and prior to 1 January 2019, with censoring at the event of interest, 90 years of age, death, or loss to follow-up (0.8%). Separate analyses restricted age-at-risk to ≥65 years to facilitate comparison with previous studies applying this minimum age threshold to address potential misclassification. Subgroup analyses compared associations of prior exposure across age-at-risk groups and across strata of baseline characteristics. Chi-squared tests for heterogeneity and for trend were applied to the stratum-specific log HRs, using inverse-variance weights. The heterogeneity test assessed whether estimates differed between strata, while the trend test assessed evidence of a linear gradient across ordered strata.^33^

Secondary analyses estimated HRs for ‘baseline’ and ‘incident’ exposures separately, adjusting for the same baseline covariates. The HRs of baseline SBP and RPG (excluding participants with prior history of diabetes) were estimated across baseline groups (SBP: <120, 120–134, 135–154, 155–179, ≥180 mmHg; RPG: <5.3, 5.3–5.8, 5.9–6.8, 6.9–7.7, ≥7.8 mmol/L) chosen to ensure clinically relevant thresholds and reasonable participant numbers in all groups and corrected for regression dilution bias using Rosner's regression method to estimate associations with usual SBP and RPG.^34^ A likelihood ratio test investigated potential departure from linearity. The 95% CIs for each group-specific log HR were estimated allowing for comparisons between any two groups.^35^

### Sensitivity analyses

Baseline associations within each of three follow-up periods (<5, 5–9, 10+ years) were explored to investigate potential reverse causality, as well as associations of SBP after excluding participants with self-reported hypertension at recruitment and, separately, participants who reported taking blood pressure lowering medications. Associations of treated vs untreated and, separately, self-reported vs screen-detected hypertension and diabetes at recruitment were compared. The effect of introducing time lags of one, two and three years between incident exposure and outcome was examined to investigate potential diagnostic bias. In order to investigate potential selection bias, we excluded incident dementia and brain atrophy identified through the clinical adjudication process. Separate analyses excluded participants from Harbin due to its overrepresentation in cases of incident brain atrophy.

All statistical analyses were done using R (version 4.3.2).

### Ethics approval

Ethical approval was obtained from the Ethical Review Committee of the Chinese Centre for Disease Control and Prevention (Beijing, China, 2004; Reference: 005/2004) and the Oxford Tropical Research Ethics Committee, University of Oxford (UK, 2005; Reference: 025-04).

### Role of the funding source

The funders of the study had no role in study design, data collection, data analysis, data interpretation, or writing of the report. The corresponding authors had full access to all data in the study and had final responsibility for the decision to submit for publication.

## Results

### Participant characteristics

Among 512,722 CKB participants included in the present analyses (eFig. 1), mean (SD) age was 52.0 (10.6), 59.0% were females, and 44.3% resided in urban regions (Table 1). Males had slightly higher SBP (132.4 [19.0] mmHg) and engaged in more physical activity (22.3 [13.4] MET-hours/day) than females (130.8 [20.5] mmHg and 20.1 [10.5] MET-hours/day, respectively). Males were also much more likely to be regular smokers (74.4% vs 3.4%) or alcohol drinkers (37.2% vs 2.5%) than females.

**Table 1:** Baseline characteristics of China Kadoorie Biobank study participants.

	Male	Female	All
**Number of participants**	210,204	302,518	512,722
**Age and socioeconomic factors**			
Age, years	52.9 (10.9)	51.4 (10.4)	52.0 (10.6)
Married	196,079 (93.3%)	266,827 (88.2%)	463,982 (90.5%)
≥6 years of education	124,044 (59.0%)	126,478 (41.8%)	250,621 (48.9%)
Household income ≥20,000 yuan	96,274 (45.8%)	123,929 (41.0%)	220,177 (42.9%)
Urban residence	91,276 (43.4%)	136,305 (45.1%)	226,951 (44.3%)
**Anthropometry, blood pressure, glucose, and lifestyle**			
BMI, kg/m^2^	23.5 (3.1)	23.8 (3.4)	23.7 (3.2)
SBP, mmHg	132.4 (19.0)	130.8 (20.5)	131.3 (19.8)
DBP, mmHg	79.2 (11.2)	76.9 (10.8)	77.9 (10.9)
RPG, mmol/L	6.0 (2.3)	6.2 (2.4)	6.1 (2.3)
Ever regular smoker	156,406 (74.4%)	10,253 (3.4%)	166,928 (32.6%)
Ever regular alcohol consumption	78,114 (37.2%)	7702 (2.5%)	85,858 (16.7%)
Physical activity, MET-h/day	22.3 (13.4)	20.1 (10.5)	21.0 (11.8)
Healthy diet score^a^	3.6 (1.1)	3.6 (1.1)	3.6 (1.1)
**Medical history and health status**			
Hypertension^b^	74,796 (35.6%)	102,929 (34.0%)	176,971 (34.5%)
Diabetes^b^	11,731 (5.6%)	19,400 (6.4%)	30,802 (6.0%)
IHD	5494 (2.6%)	10,284 (3.4%)	15,746 (3.1%)
Stroke/TIA	4720 (2.2%)	4247 (1.4%)	9049 (1.8%)
Medications			
Statin	445 (0.2%)	717 (0.2%)	1167 (0.2%)
Antihypertensive	21,806 (10.4%)	38,261 (12.6%)	59,814 (11.7%)
Glucose lowering	3229 (1.5%)	5966 (2.0%)	9069 (1.8%)
Poor self-reported health	18,642 (8.9%)	34,902 (11.5%)	53,392 (10.4%)

Data presented are mean (SD) or N (%). Standardized for age, sex and study area, as appropriate.

BMI, body mass index; MET, metabolic equivalent of task; SBP, systolic blood pressure; DBP, diastolic blood pressure; RPG, random plasma glucose; IHD, ischaemic heart disease; TIA, transient ischaemic attack.

a Healthy diet score (0–9): 1 point for each of the following components: daily fresh vegetables, ≥4 days/week fresh fruit, soy foods, eggs, other stable foods, ≥1 day/week fish, 1–6 days/week red meat, dairy, <4 days/week preserved vegetables.

b Self-reported or detected by screening at baseline.

At recruitment, 34.5% of participants reported a diagnosis of hypertension (11.9%) or had screen-detected hypertension (22.6%). Diabetes was self-reported or screen-detected in 6.0% (3.2% self-reported; 2.8% screen-detected) of participants. IHD was reported by 3.1% and stroke/TIA was reported by 1.8%. By the end of follow-up on 1 January, 2019 (median 12.1 [IQR 11.1–13.1] years), 26,652 participants were newly diagnosed with hypertension, 23,747 with diabetes, 56,504 with IHD, and 64,744 with stroke.

### Distribution of dementia and brain atrophy

During approximately 6 million person-years of follow-up, 56,549 (11.0%) participants died and 4028 (0.8%) were lost to follow-up. A total of 1099 incident dementia cases (173 vascular, 344 Alzheimer's, 646 other) and 1471 brain atrophy cases were recorded at age-at-risk ≥40 years.

**Fig. 1:**
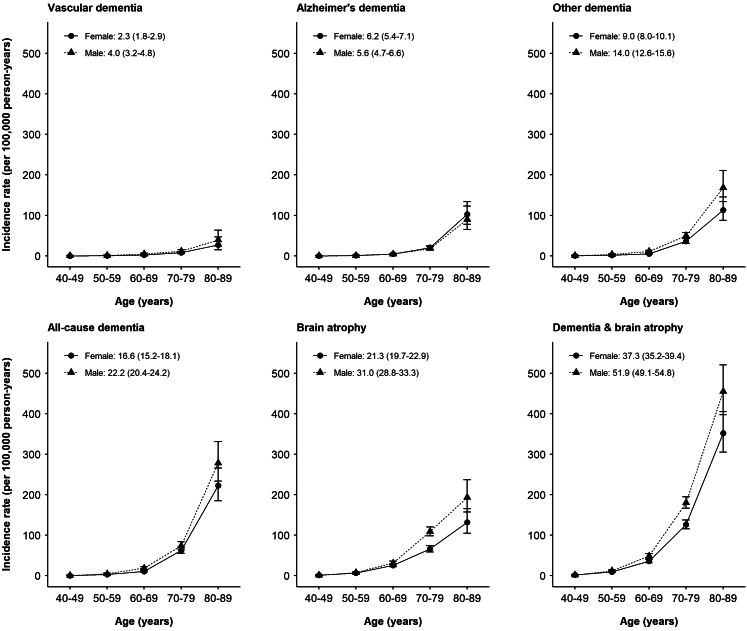
**Dementia and brain atrophy incidence rates, by age and sex**. Sex-specific region-standardised incidence rates. Vertical lines represent 95% CIs.

The age- and sex-standardised incidence rates (per 100,000 person-years) were 19.0 (95% CI 17.9–20.1) for ACD, 3.0 (2.6–3.5) for VD, 5.9 (5.3–6.6) for AD, 11.2 (10.3–12.1) for other dementia, and 25.4 (24.1–26.8) for brain atrophy. Incidence rates increased exponentially with age, rising from 2.3 (1.8–2.8) per 100,000 person-years for ACD and 4.4 (3.7–5.1) for brain atrophy in those under 60 years, to 230.7 (204.3–259.6) and 170.7 (148.1–195.9), respectively, for those aged 80–89 years, with qualitatively similar trends observed for dementia subtypes (Fig. 1).

Vascular and other dementia incidence rates were higher in males and rural regions, whereas AD incidence rates showed no clear differences by sex or region (Fig. 1, eFig. 2). Incidence rates for brain atrophy were higher in males and urban regions, although urban-rural differences were largely driven by one urban region (Harbin) which contributed half of cases (n = 713; eFig. 3). There were no differences in dementia or brain atrophy incidence rates by education level (eFig. 4). Most participants (96%) with a diagnosis of dementia or brain atrophy had just one dementia subtype (or brain atrophy) recorded during follow-up (eFig. 5).

### Cardiometabolic health and risk of dementia and brain atrophy

**Fig. 2:**
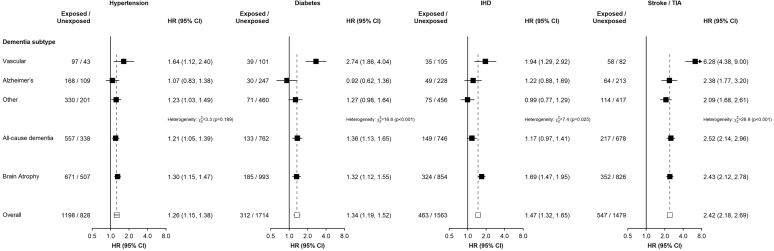
**Associations of markers of prior cardiometabolic health with risk of dementia and brain atrophy**. The hazard ratios (HRs) are shown as squares (not scaled proportional to the variance of the log risk) and 95% CIs are shown as horizontal lines. The chi-square and p-values are shown for heterogeneity between dementia subtypes. HRs were stratified by age-at-risk (5-year age groups), sex and region and adjusted for smoking, alcohol consumption, education, physical activity, healthy diet score, and BMI. Exposure is self-reported or screen-detected at recruitment or the first instance of the exposure identified by ICD-10 codes recorded at hospitalisation or in disease registries at least one year prior to incident dementia or brain atrophy. BMI, body mass index; IHD, ischaemic heart disease; TIA, transient ischaemic attack.

After controlling for sociodemographic and lifestyle factors and BMI, any prior (i.e. recorded at baseline or during follow-up) hypertension (adjusted HR 1.21 [95% CI 1.05–1.39]), diabetes (1.36 [1.13–1.65]), and stroke/TIA (2.52 [2.14–2.96]) were all positively associated with risk of ACD (Fig. 2). The association for IHD was less clear (1.17 [0.97–1.41]). All exposures were most strongly associated with VD (hypertension: 1.64 [1.12–2.40]; diabetes: 2.74 [1.86–4.04]; IHD: 1.94 [1.29–2.92]; stroke/TIA: 6.28 [4.38–9.00]) compared to other subtypes or brain atrophy. For non-vascular dementias, there was a clear positive association of stroke/TIA, with HRs of 2.38 (1.77–3.20) and 2.09 (1.68–2.61) for AD and other dementia, respectively. Hypertension was associated weakly with other dementia (1.23 [1.03–1.49]) and less clearly with AD (1.07 [0.83–1.38]), but tests of heterogeneity showed no apparent differences by dementia subtype (*p* = 0.189). IHD (1.22 [0.88–1.69] and 0.99 [0.77–1.29], for AD and other dementia, respectively) and diabetes (0.92 [0.62–1.36] and 1.27 [0.98–1.64], respectively) were not associated with non-vascular dementia. All prior exposures showed strong positive associations with brain atrophy (hypertension: 1.30 [1.15–1.47]; diabetes: 1.32 [1.12–1.55]; IHD: 1.69 [1.47–1.95]; stroke/TIA: 2.43 [2.12–2.78]).

**Fig. 3:**
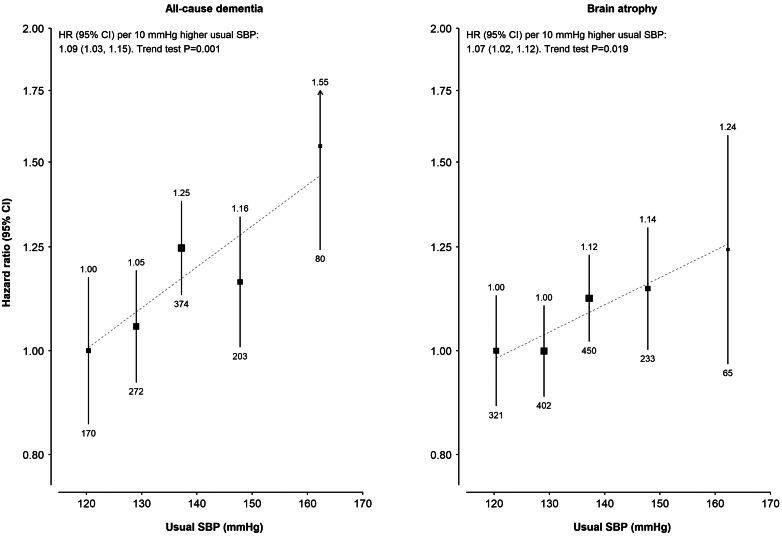
**Association of usual SBP with risk of all-cause dementia and brain atrophy**. Each square represents the adjusted hazard ratio (HR) with the area inversely proportional to the variance of the category specific log HR. HRs are plotted on a floating absolute risk scale. Vertical lines indicate 95% CIs. The HRs are shown above each square and numbers of events below. Chi-squared tests were applied to the stratum-specific log HRs, using inverse-variance weights to assess evidence of a linear gradient across ordered strata. The regression dilution ratio (RDR = 0.50) for estimating usual SBP values was calculated using Rosner's regression method adjusting for region, sex, and 10-year age group. HRs are plotted against the mean usual SBP in each group, estimated by (B–A) x RDR + A, where A is the overall baseline mean SBP, and B is the baseline SBP group mean.

There were positive log–linear associations between usual SBP and risk of ACD and brain atrophy (Fig. 3). Each 10 mmHg higher usual SBP was associated with a HR of 1.09 (95% CI 1.03–1.15) for ACD and 1.07 (1.02–1.12) for brain atrophy, with no evidence of departure from linearity (*p* > 0.05). Analyses by dementia subtype yielded results similar to those for ACD (eFig. 6). Exclusion of participants who reported taking blood pressure lowering medications (11.5%) at baseline did not alter these findings (data not shown). Among participants without a prior history of diabetes at recruitment, there were higher risks of VD at the highest RPG category (≥7.8 mmol/L), but no clear associations at lower levels or with other dementia subtypes or brain atrophy (eFigs. 7 and 8).

Associations of prior diabetes with ACD (*p*_trend_ = 0.006) and of stroke/TIA with ACD (*p*_trend_ ≤0.001) and with brain atrophy (*p*_trend_ ≤0.001) were stronger at younger ages. The positive associations between prior diabetes (*p*_heterogeneity_ = 0.001) and stroke (*p*_heterogeneity_ = 0.008) with brain atrophy were stronger in rural regions (eFig. 9–12). Restricting the analyses to dementia and brain atrophy with onset at ≥65 years weakened the association between stroke and VD (4.47 [2.99–6.67] vs 6.28 [4.38–9.00]), but otherwise did not materially alter the findings (eFig. 13). In addition, the results were broadly similar after restricting incident dementia and brain atrophy to cases identified via linkage to national health registries and insurance databases (data not shown).

Compared to baseline associations, time-dependent analyses of new-onset exposures (with one year lag) yielded stronger associations with brain atrophy (hypertension: HR 1.90 [95% CI 1.50–2.40] vs 1.20 [1.08–1.34]; diabetes: 1.92 [1.46–2.52] vs 1.16 [0.98–1.37]; IHD: 2.07 [1.76–2.43] vs 1.19 [1.00–1.42]; and stroke: 2.73 [2.36–3.17] vs 1.31 [1.05–1.63] for incident and baseline associations, respectively; eFig. 14) but had no clear effect on associations with ACD. Introducing longer time lags between incident exposure and outcome attenuated the associations with brain atrophy; nevertheless, the association with incident stroke remained marginally stronger than the baseline association even after excluding up to three years following an incident event (1.82 [1.39–2.38]; eFig. 14). Excluding Harbin did not notably alter associations with brain atrophy (eFig. 15). Associations with baseline exposures were similar across different durations of follow-up (eFig. 16), and did not vary substantially when comparing self-reported and screen-detected, or treated and untreated hypertension or diabetes (eFig. 17).

## Discussion

This large prospective study of over 0.5 million Chinese adults showed that markers of cardiometabolic health were positively associated with risk of dementia overall and with major subtypes, particularly VD. Similar associations were found with brain atrophy and associations remained after controlling for the potential influence of diagnostic bias or reverse causality. The present findings are particularly noteworthy given the high burden of vascular disease in this Chinese population; over the study period, approximately one in eight participants was diagnosed with stroke and/or IHD.

Stroke/TIA emerged as the strongest risk factor for dementia, associated with a more than doubling of risk. A previous meta-analysis reporting on the associations of prevalent (36 studies, ∼1.9 million participants) and incident (12 studies, ∼1.3 million participants) stroke with ACD reported pooled HRs of 1.69 (95% CI 1.49–1.92) and 2.18 (1.90–2.50), respectively, with somewhat higher risks among males.^36^ These risk estimates, although slightly weaker, are consistent with our findings. We likewise found a possibly stronger association of incident (HR 2.49) compared to prevalent (HR 2.21) stroke, even after adding a one-year gap between exposure and outcome, and some weak evidence of a stronger association in males. We examined whether scanning or diagnostic bias may be influencing the strength of associations in CKB, particularly for incident stroke and dementia. However, sensitivity analyses suggested that these results were robust and showed no attenuation over longer windows of follow-up after excluding events in the first year after incident stroke. Additionally, stroke/TIA was independently associated with higher risk of all dementia subtypes. The association with AD (2.38 [1.77–3.20]) in the present analyses is stronger than in a recent meta-analysis of predominantly Western populations (1.40 [1.06–1.85]) and in an East Asian population (1.79 [1.28–2.50]), possibly reflecting underestimation of the strength of association in the meta-analysis due to short follow-up and attrition bias.^37^^,^^38^ Alternatively, the ability to account for incident stroke in CKB—reducing exposure misclassification—may have contributed to the stronger association. Overall, these findings confirm stroke/TIA as a key dementia risk factor in China and emphasise the need for targeted preventive interventions aimed at mitigating long-term cognitive decline following disability-free stroke/TIA.^39^

The present study found positive associations of hypertension, diabetes, and IHD with risk of dementia. Several meta-analyses of prospective studies have reported on these associations,^40^, ^41^, ^42^, ^43^, ^44^, ^45^, ^46^, ^47^ including one large individual participant data meta-analysis incorporating data from 17 studies (N = 34,519; mean age: 72.5; follow-up: 4.3 years).^40^ Overall, these meta-analyses reported positive associations of hypertension (HRs 1.02–1.42; depending on mid-life/late-life, and treated/untreated exposure),^40^^,^^41^ diabetes (HRs 1.43–1.68),^42^, ^43^, ^44^, ^45^ and IHD (1.26–1.45)^46^^,^^47^ with ACD. In CKB, these associations were driven almost entirely by strong associations with VD, suggesting robust dementia classification and mirroring results from the studies that examined VD separately.^41^, ^42^, ^43^, ^44^, ^45^ These findings highlight the importance of effective control of hypertension in this population with reported high prevalence of undiagnosed and uncontrolled hypertension (∼70% and ∼95%, respectively, at recruitment in CKB)^19^ and of both population- and individual-level interventions to address this.^17^^,^^48^ However, we found no association between diabetes or IHD and non-vascular dementias, contrasting with positive associations reported in previous meta-analyses,^37^^,^^42^, ^43^, ^44^ and in three large prospective cohort studies (which accounted for 99% of East Asian data in the meta-analyses described above).^38^^,^^49^^,^^50^ These previous East Asian studies differ importantly from CKB; all relied exclusively on health registry data and were unable to account for socioeconomic and lifestyle factors with likely residual confounding, two included relatively short follow-up periods^38^^,^^50^ and two investigated only baseline exposures.^49^^,^^50^

In contrast with the clear positive association of hypertension and blood pressure with VD, the relevance of hypertension as a risk factor for non-vascular dementia was mixed in CKB, with no apparent association of prior hypertension or usual SBP with AD, but a modest association with other dementia. These findings are consistent with null or inconclusive associations between hypertension and non-vascular dementias reported in meta-analyses and prospective East Asian studies.^13^^,^^37^^,^^38^^,^^41^^,^^49^, ^50^, ^51^, ^52^ Moreover, given elevated blood pressure is a well-established risk factor for stroke^6^^,^^53^ which, in turn, is strongly associated with risk of non-vascular dementia, modest associations of hypertension with the category of other dementia could reflect confounding by stroke.

Although CKB was not designed to be nationally representative, it is notable that the incidence rates of dementia in CKB (19.0 per 100,000 person-years) were substantially lower than national estimates for China derived from the Global Burden of Disease Study 2021, which reported rates of 151.4 per 100,000.^4^ This difference may reflect the age range of participants, restriction of recruitment in CKB to community-based disability-free populations, and reliance on hospitalisation and mortality data for follow-up. Underdiagnosis and misdiagnosis of dementia likely also contribute, reflecting a range of factors, including stigma associated with dementia in China and healthcare factors.^54^ Although not unique to China, these considerations highlight the challenges of achieving accurate estimates of dementia incidence in this and other populations.

Clinical reports of brain atrophy, although not synonymous with dementia, may serve as a proxy of brain health, particularly when underreporting of dementia is suspected. The widespread recommended use of brain imaging in China (e.g. as a routine investigation for patients with cognitive impairment, motor, bowel, and affective disorders following exclusion of other organic diseases^15^) facilitated investigation of brain atrophy in the present study.^20^ To the best of our knowledge, no previous epidemiological study has examined risk factors for brain atrophy captured by clinical reports. However, evidence from imaging studies strongly suggests that vascular risk factors and vascular incidents are inversely associated with brain volume and other imaging indices.^55^, ^56^, ^57^, ^58^, ^59^, ^60^, ^61^ The present analyses demonstrated that the prospective associations of markers of cardiometabolic health with brain atrophy mirror those seen with dementia, warranting further investigation to understand the clinical relevance and generalisability of these findings.

This study has several strengths. It is the first large-scale prospective cohort study in mainland China to explore the relationship between markers of cardiometabolic health and incident dementia, and the first in any population to explore associations with clinically reported brain atrophy. CKB provides long-term, systematic follow-up of disease outcomes in community-dwelling, relatively healthy adults, with <1% lost to follow-up, and outcome adjudication to improve diagnostic accuracy, minimising the risk of reverse causality and selection bias and allowing associations to be explored by different periods of follow-up. Moreover, the robust exposure measurement, which included previously diagnosed and screen-detected prevalent exposures combined with incident exposures recorded during follow-up, reduced misclassification. Furthermore, resurvey data allowed for assessing usual SBP and RPG levels through correction for regression dilution bias. Nevertheless, our study also has limitations. The relatively young mean age at recruitment of community-based participants and limited follow-up data from primary or institutionalised care settings may have contributed to relatively low overall dementia incidence rates, particularly for AD and VD. This may also reflect some misdiagnosis or underdiagnosis of dementia (adjudication of these endpoints is not yet complete in CKB) which would be expected to result in underestimation of the strength of associations of markers of cardiometabolic health with risk of dementia. This has implications for generalisability of the study findings to other, including older or institutionalised, cohorts. Moreover, it resulted in reduced statistical power to further explore subgroup-specific associations, including by age, for example, to examine risk of young-onset dementia, sex and ApoE status or genetic predisposition to dementia. Future work using whole-genome sequencing data will contribute insights into these latter aspects and potential mechanisms underlying identified associations. We were also unable to explore other markers of cardiometabolic health (e.g. lipids) which may be related to dementia onset. Likewise, while the cardiometabolic risk factors adjusted for in our model, (e.g. adiposity, physical activity) have also been suggested to be associated with higher dementia risk,^5^ because these exposures are particularly prone to confounding and reverse causality given the long prodromal stage of dementia, longer follow-up would be required to address these potential risk factors reliably.^62^ Finally, we were unable to distinguish between physiological and pathological brain atrophy, which may differ in their underlying causes, symptoms, and impact on, for example, cognitive function.

In conclusion, our study shows that hypertension, diabetes, IHD and stroke are adversely associated with brain health in later life. In contrast to weak evidence for the associations of diabetes and blood pressure with non-vascular dementias, stroke incidence was strongly associated with all dementia subtypes and, in particular, VD. Our findings reinforce established associations between markers of cardiometabolic health and dementia, providing new evidence from an understudied population. Given the high rates and strikingly poor long-term prognosis of stroke in China,^63^ this study highlights the added importance of controlling intermediate risk factors, such as blood pressure, for preventing stroke and maintaining brain health.

## Contributors

C.B.L. contributed to the concept and design of the study, conducted the statistical analyses and drafted the manuscript. A.I. and F.B. contributed to the concept and design of the study, drafted the manuscript and provided critical revision of the manuscript for important intellectual content. D.A. and I.T. provided administrative, technical or material support. Y.C. and L.L. were involved in the acquisition of data, obtained funding and provided critical revision of the manuscript for important intellectual content. L.Y., H.D., J.C., P.P., J.L., C.Y., D.S., and N.Z. provided critical revision of the manuscript for important intellectual content. Z.C. obtained funding, contributed to the concept and design of the study, provided critical revision of the manuscript for important intellectual content, and, together with N.Z., was involved in the acquisition of the data. C.B.L, A.I., F.B. and Z.C. are the guarantors of this work and, as such, they had full access to all the data in the study and take responsibility for the integrity of the data and the accuracy of the data analysis. There was no use of artificial intelligence (AI) tools.

## Data sharing statement

Data from baseline, first and second resurveys, and disease follow-up are available for access by *bona fide* researchers. Details of the China Kadoorie Biobank Data Sharing Policy and procedures for data access are available at www.ckbiobank.org.

## Open access statement

For the purpose of Open Access, the author has applied a CC-BY public copyright license to any Author Accepted Manuscript version arising from this submission.

## Ethics approval

The China Kadoorie Biobank complies with all the required ethical standards for medical research on human subjects. Ethical approvals were granted and have been maintained by the relevant institutional ethical research committees in the UK and China.

## Consent to participate/publication

All participants provided written informed consent.

## Declaration of interests

CvD reports other financial or non-financial interests with NovoNordisk and GSK. All other authors declare no competing interests.
