# Supplementary material for: Cardiometabolic health and risk of dementia and brain atrophy: a community-based prospective cohort study of 0.5 million adults in China
Source: Lancet Reg Health West Pac. 2025 Nov 18;64:101743. doi: 10.1016/j.lanwpc.2025.101743 (PMC12670887; doi:10.1016/j.lanwpc.2025.101743)
Supplement: Supplemental Material [file mmc1.pdf]

Supplementary Material Content

Cardiometabolic health and risk of dementia and brain atrophy: a community-based prospective cohort study of 0.5 million adults in China

China Kadoorie Biobank collaborative group and members 2

eTable 1: Definitions of cardiometabolic exposures and disease outcomes 4

eFigure 1: Flowchart of CKB participant selection and study design 5

eFigure 2: Dementia and brain atrophy incidence rates, by age and urban/rural residence ... 6

eFigure 3: Dementia and brain atrophy incidence rates, by study region 7

eFigure 4: Dementia and brain atrophy incidence rates, by age and education 8

eFigure 5: Number of participants with diagnoses of single or multiple incident dementia subtypes and brain atrophy 9

eFigure 6: Association of usual SBP with risk of dementia subtypes 10

eFigure 7: Association of usual RPG with risk of all-cause dementia and brain atrophy, among participants without a prior history of diabetes at recruitment 11

eFigure 8: Association of usual RPG with risk of dementia subtypes, among participants without a prior history of diabetes at recruitment 12

eFigure 9: Association of hypertension with risk of all-cause dementia and brain atrophy, by baseline characteristics 13

eFigure 10: Association of diabetes with risk of all-cause dementia and brain atrophy, by baseline characteristics 14

eFigure 11: Association of IHD with risk of all-cause dementia and brain atrophy, by baseline characteristics 15

eFigure 12: Association of stroke/TIA with risk of all-cause dementia and brain atrophy, by baseline characteristics 16

eFigure 13: Associations of markers of prior cardiometabolic health with risk of dementia and brain atrophy at age-at-risk ≥65 years 17

eFigure 14: Associations of markers of cardiometabolic health with risk of dementia and brain atrophy after introducing various time lags between exposure diagnosis and outcomes 18

eFigure 15: Associations of markers of prior cardiometabolic health with risk of brain atrophy, excluding participants from Harbin 19

eFigure 16: Associations of markers of cardiometabolic health at recruitment with risk of all-cause dementia and brain atrophy, by duration of follow-up 20

eFigure 17: Associations of hypertension and diabetes subgroups at recruitment with risk of all-cause dementia and brain atrophy 21

China Kadoorie Biobank collaborative group and members

International Steering Committee: Junshi Chen, Zhengming Chen (PI), Robert Clarke, Rory Collins, Liming Li (PI), Jun Lv, Richard Peto, Robin Walters.

International Co-ordinating Centre, Oxford: Daniel Avery, Maxim Barnard, Derrick Bennett, Ruth Boxall, Ka Hung Chan, Yiping Chen, Zhengming Chen, Jonathan Clarke; Robert Clarke, Huaidong Du, Ahmed Edris Mohamed, Hannah Fry, Simon Gilbert, Prapthi Harish, Pek Kei Im, Andri Iona, Christiana Kartsonaki, Kshitij Kolhe, Hubert Lam, Kuang Lin, James Liu, Iona Millwood, Sam Morris, Qunhua Nie, Alfred Pozarickij, Maryam Rahmati, Paul Ryder, Dan Schmidt, Becky Stevens, Iain Turnbull, Robin Walters, Baihan Wang, Lin Wang, Neil Wright, Ling Yang, Xiaoming Yang, Pang Yao.

National Co-ordinating Centre, Beijing: Jun Lv, Canqing Yu, Dianjianyi Sun, Yuanjie Pang, Can Hou, Qingmei Xia, Chao Liu, Pei Pei, Lang Pan, Xiao Han, Honglu Bian, Xinxin Chen.

10 Regional Co-ordinating Centres: Qingdao CDC: Zengchang Pang, Ruqin Gao, Shanpeng Li, Haiping Duan, Shaojie Wang, Yongmei Liu, Ranran Du, Liang Cheng, Xiaocao Tian, Hua Zhang. **Licang CDC:** Dan Hu, Xiaoyan Zheng, Yujie Wang. **Heilongjiang Provincial CDC:** Wei Sun, Shichun Yan, Xiaoming Cui. **Nangang CDC:** Chi Wang, Zhenyuan Wu, Lishun Zhai, Zhaoxi Pang, Shiwen Dong. **Hainan Provincial CDC:** Huiming Luo, Jinyan Chen, Bin He, Dingwei Sun, Xingren Wang, Tingting Ou. **Meilan CDC:** Xiangyang Zheng, Dewei Zheng, Shuai Yang, Yilei Li, Lihui Li, Xingjiao Chen. **Jiangsu Provincial CDC:** Jinyi Zhou, Ran Tao, Jian Su, Xikang Fan, Zongming Cheng, Yuxiao Huang. **Suzhou CDC:** Yan Lu, Yujie Hua, Li Xing, Shuxian Wang, Jianrong Jin, Juping Ma, Jinchao Liu, Kaifei Zhu, Hongfu Ren, Xingfeng Shen. **Guangxi Provincial CDC:** Ge Zhong, Wei Mao, Zhenzhen Lu, Ling He. **Liuzhou CDC:** Lifang Zhou, Changping Xie, Jian Lan, Tingping Zhu, Jinxue Tan, Liuping Wei, Liyuan Zhou, Sisi Wang. **Sichuan Provincial CDC:** Xianping Wu, Ningmei Zhang, Xiaofang Chen, Xiaoyu Chang, Zhuo Wang, Yujin He. **Pengzhou CDC:** Mingqiang Yuan, Xia Wu, Xiaofang Chen, Zhaodong Wang, Qiang Sun, Yang Lin. **Gansu Provincial CDC:** Faqing Chen, Xiaolan Ren, Lijun Chang, Feiming Zhong. **Maiji CDC:** Jianjun Feng, Weijie Hu, Xiaofang Zhang, Yalin Chen, Fei Wang, Jun Wang. **Henan Provincial CDC:** Linqi Diao, Wanshen Guo, Zhiwei Han, Dongyang Zhao, Dengjun Zhu, Kai Kang, Shixian Feng, Huizi Tian, Yali Yan, Bing Han, Li Gao, Shaofang Li, Huafei Feng, Wei Tang. **Huixian CDC:** Xiaolin Li, Huarong Sun, Xiaocong Zhao, Ying Li, Chen Hu, Pan He, Xukui Zhang, Yuanyuan Jin, Hesheng Zhang. **Zhejiang Provincial CDC:** Min Yu, Ruying Hu, Hao Wang, Weiwei Gong, Jieming Zhong, Meng Wang, Chunxiao Xu, Keqing Gong. **Tongxiang CDC**: Hao Xu, Yuan Cao, Kaixu Xie, Lingli Chen, Xiaomei Tu,Chen Chen. **Hunan Provincial CDC:** Xiaojun Li, Li Yin, Huilin Liu, Yuan Liu, Yi Liu, Lei Yin, Xian Xie, Jing Wang. **Liuyang CDC:** Bo Xiao, Pingsheng Lou, Yuan Peng, Libo Zhang, Chan Qu, Qili Jiang, Yanling Chen, Yan Zhao.

Event Adjudication Clinicians: Beijing Tiantan Hospital,Capital Medical University Shuya Li, Haiqiang Qin, Yongjun Wang, **Peking University People's Hospital** Qiling Chen, Jihua Wang, **The 1st Affiliated Hospital of Harbin Medical University** Xiaojia Sun, Lei Wang, Xun Wang, Liming Zhang, Shanshan Zhou, **The 2nd Affiliated Hospital of Harbin Medical University** Hongyuan Chen, Li Chen, Haiyan Gou, Weizhi Wang, Yanmei Zhu, Yulan Zhu, **The 2nd Hospital of Hebei Medical University** Ning Zhang, **Huashan Hospital** Xin Cheng, Qiang Dong, Yi Dong, Kun Fang, Yiting Mao, **Jinling Hospital** Yu An, Peiling Chen, Yinghua Chen, Zhihong Liu, Lihua Zhang **The People's Hospital of Liaoning Province** Xiaohong Chen, Naixin Jv, Xiaojiu Li, Liyang Liu, Yun Lu, Xiaona Xing, **Qingdao Fuwai Cardiovascular Hospital** Shihao You, **Shengjing Hospital of China Medical**

University Xiaoli Cheng, Chaojun Gua, Jinping Jiang, Jingyi Liu, Shumei Ma, **Shenyang Military General Hospital** Xuefeng Yang, **The First People's Hospital of Shenyang** Xiaomo Du, Jian Xu, Xuecheng Yang, Xiaodi Zhao, **West China Hospital, Sichuan University** Zilong Hao, Ming Liu, Deren Wang, **The Second Affiliated Hospital of Suzhou University** Xiaoting Li, **Suzhou Kowloon Hospital Shanghai Jiao Tong University School of Medicine** Lili Hui, Zhanling Liao, Feng Liu, **Qingdao Fuwai Cardiovascular Hospital** Chunning Feng, Dejiang Ji, Fengxia Qu, Wenwen Yuan, **The First Affiliated Hospital of Zhengzhou University** Xin Fu, **Zhongshan Hospital,** Jing Ding, Peng Du, Lirong Jin, Yueshi Mao, Xin Wang.

eTable 1: Definitions of cardiometabolic exposures and disease outcomes

	No. of first events	Definitions and ICD-10 codes
**EXPOSURES**		
**Hypertension**		
Baseline	176,971	Self-reported or SBP ≥140 mmHg or DBP ≥90 mmHg
Incident	26,652	I10-I12, O13, H35.0, I15.0, I15.1, I15.9, I67.4
**Diabetes**		
Baseline	30,802	Self-reported or RPG ≥11·1 mmol/L (<8 hours since last eating) or RPG ≥7·0 mmol/L (≥8 hours since last eating) or FPG ≥7·0 mmol/L.
Incident	23,747	E10-E14
**IHD**		
Baseline	15,746	Self-reported
Incident	56,504	I20-I25
**Stroke/TIA**		
Baseline	9,049	Self-reported
Incident	64,744	I60-I61, I63-I64
**DISEASE ENDPOINTS**		
**Dementia**		
Vascular	173	F01; F01.0; F01.1; F01.2; F01.3; F01.8; F01.9; I67.3
Alzheimer's	345	F00; F00.0; F00.1; F00.2; F00.9; G30; G30.0; G30.1; G30.8; G30.9
Other	648	F02; F02.0; F02.1; F02.2; F02.3; F02.4; F02.8; F03; F05.1; G31.0; G31.1; G31.8; A81.0; F10.6
All-cause	1,102	F01; F01.0; F01.1; F01.2; F01.3; F01.8; F01.9; I67.3; F00; F00.0; F00.1; F00.2; F00.9; G30; G30.0; G30.1; G30.8; G30.9; F02; F02.0; F02.1; F02.2; F02.3; F02.4; F02.8; F03; F05.1; G31.0; G31.1; G31.8; A81.0; F10.6
**Brain atrophy***	1,471	G31.9
**Overall**	2,520	Any ICD-10 code listed above

*Includes 53 participants with an overlapping dementia diagnosis. No. of first events for incident exposures excludes participants with the condition at baseline. SBP: systolic blood pressure; DBP: diastolic blood pressure; RPG: random plasma glucose; FPG: fasting plasma glucose; IHD: ischaemic heart disease; TIA: transient ischaemic attack.

eFigure 1: Flowchart of CKB participant selection and study design

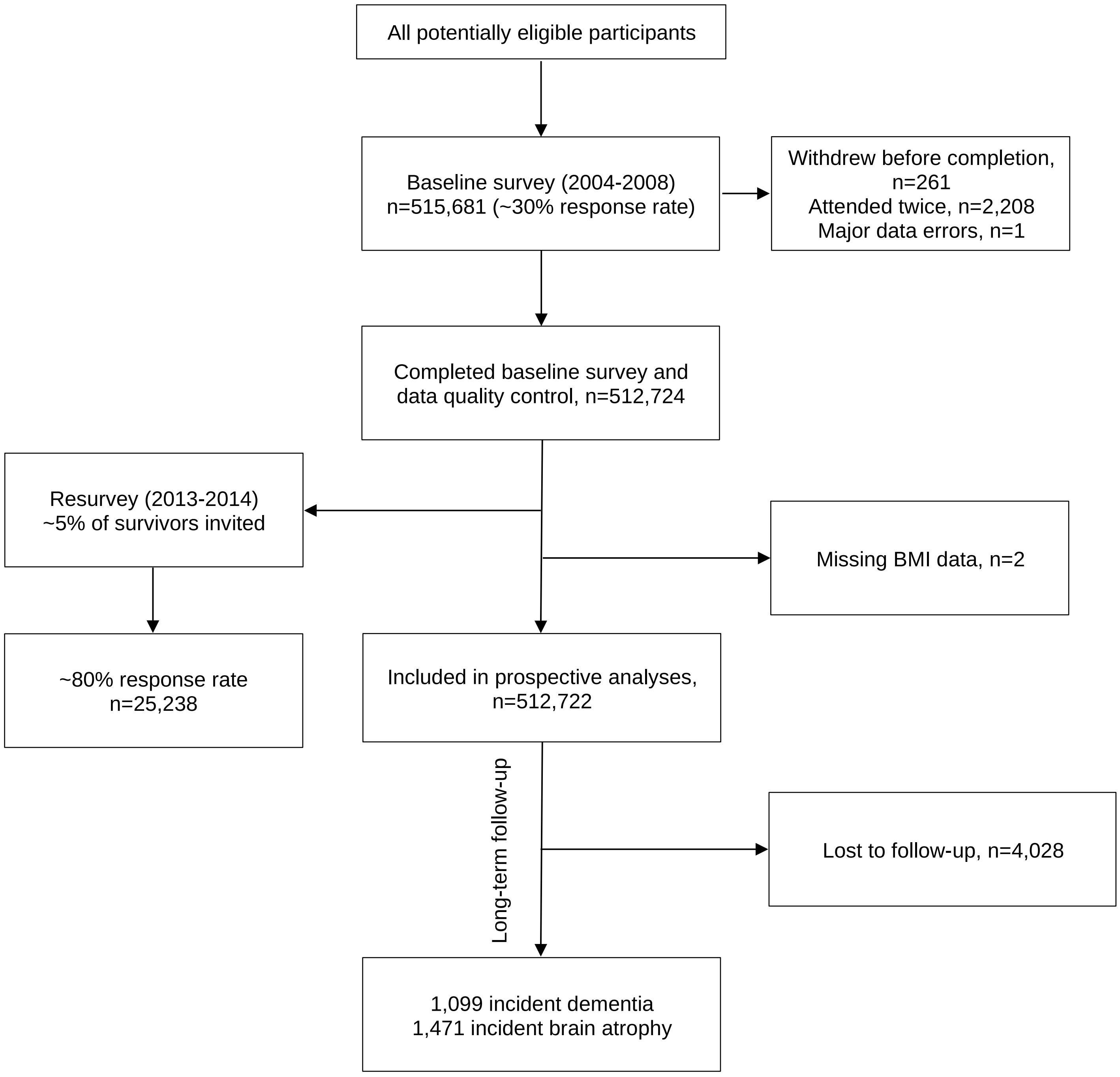

eFigure 2: Dementia and brain atrophy incidence rates, by age and urban/rural residence

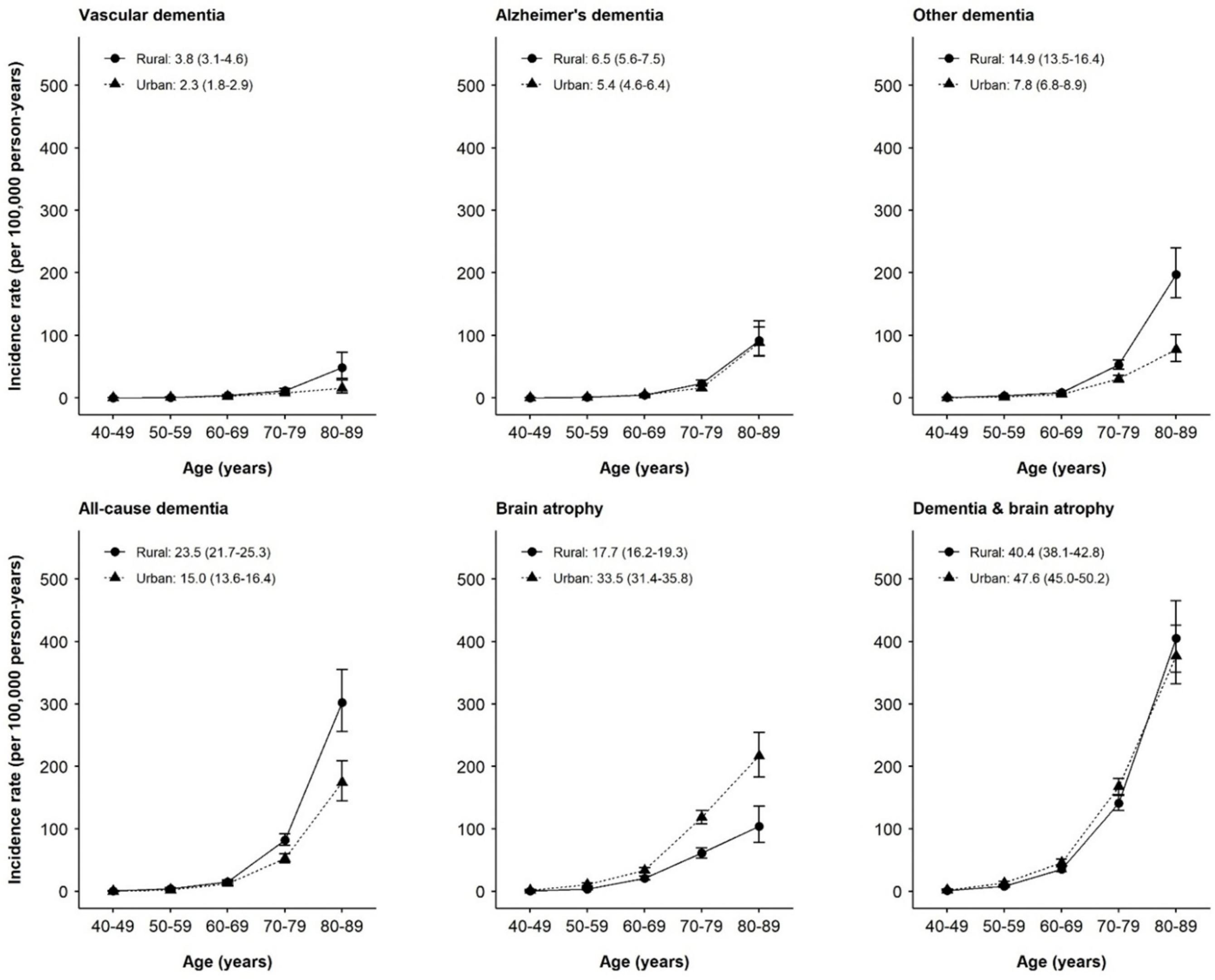

Rural- and urban-specific sex-standardised incidence rates. Vertical lines represent 95% CIs.

eFigure 3: Dementia and brain atrophy incidence rates, by study region

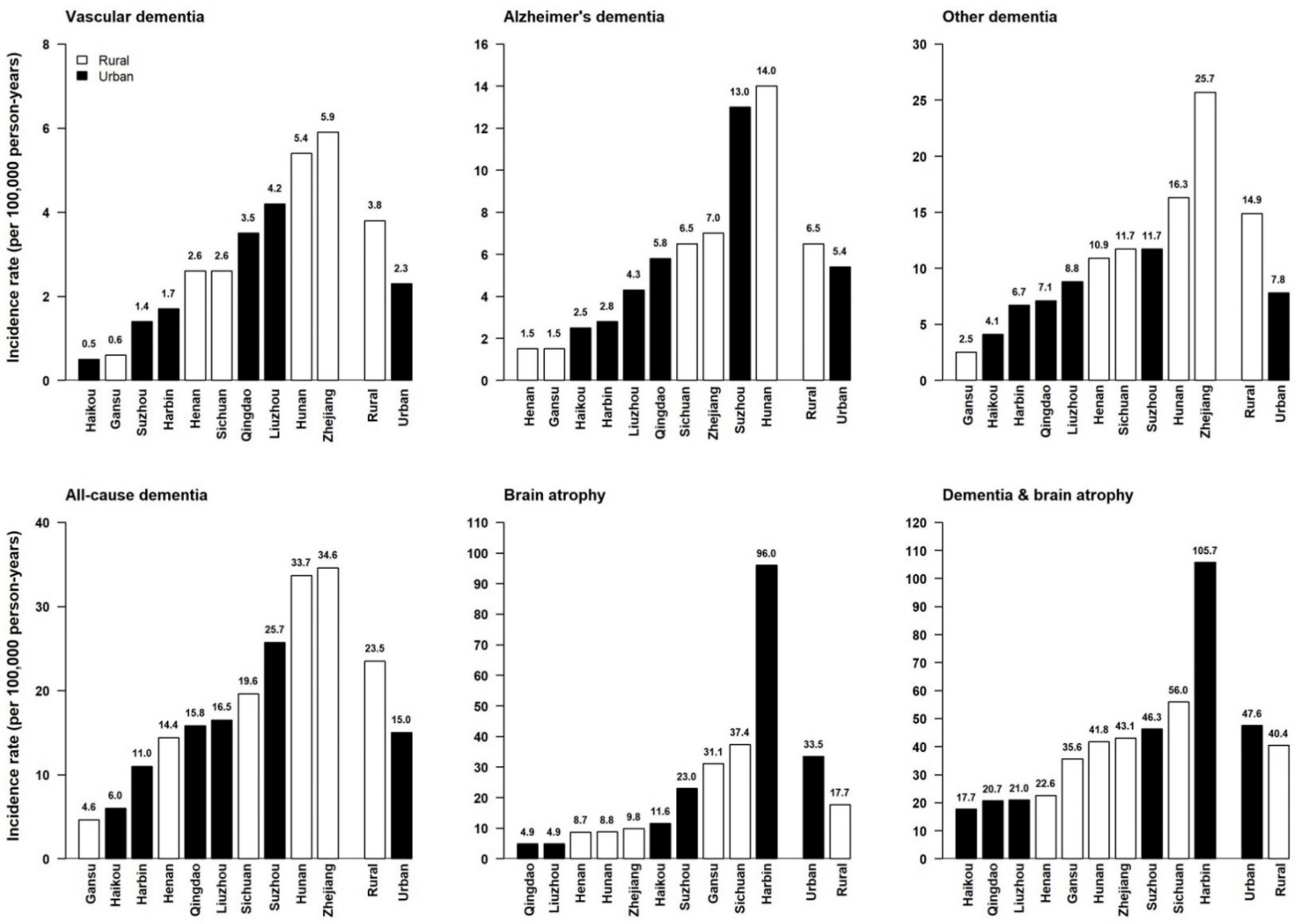

Age- and sex- standardized incidence rates.

eFigure 4: Dementia and brain atrophy incidence rates, by age and education

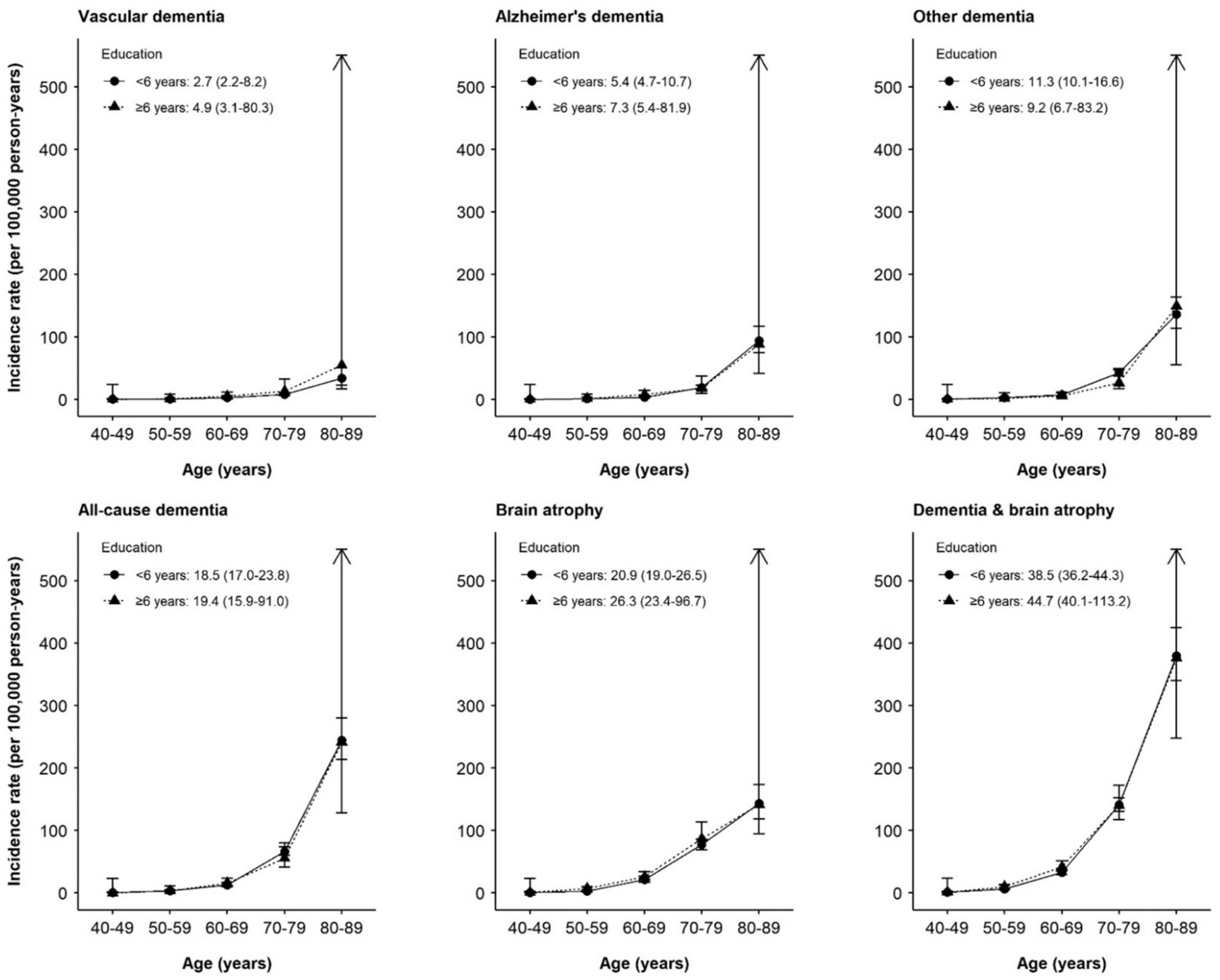

Sex- and region-standardised incidence rates. Vertical lines represent 95% CIs.

eFigure 5: Number of participants with diagnoses of single or multiple incident dementia subtypes and brain atrophy

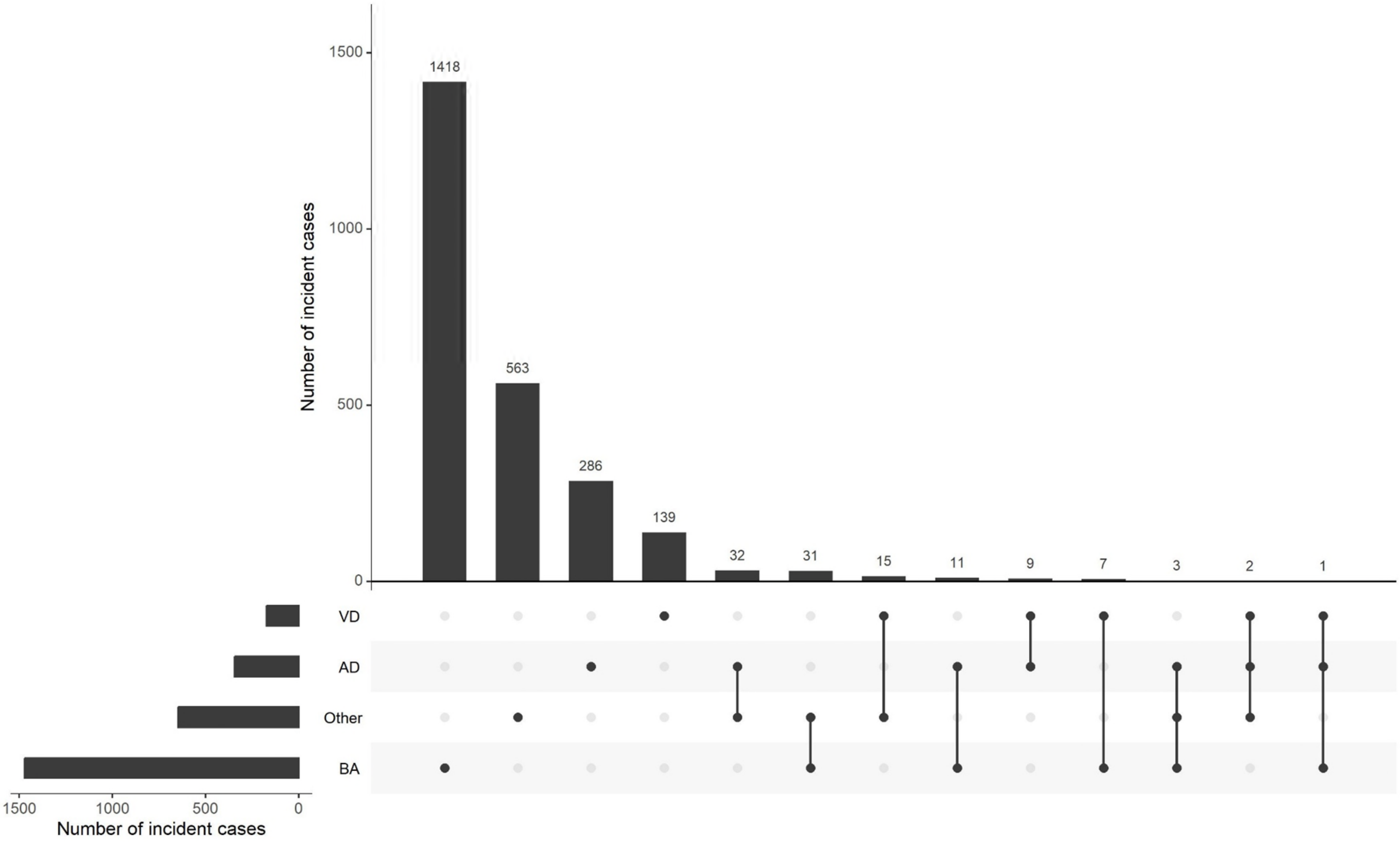

Number of incident cases of specific and mixed pathology. VD: Vascular dementia; AD: Alzheimer's dementia; Other: Other/Unspecified dementia; BA: Brain atrophy.

eFigure 6: Association of usual SBP with risk of dementia subtypes

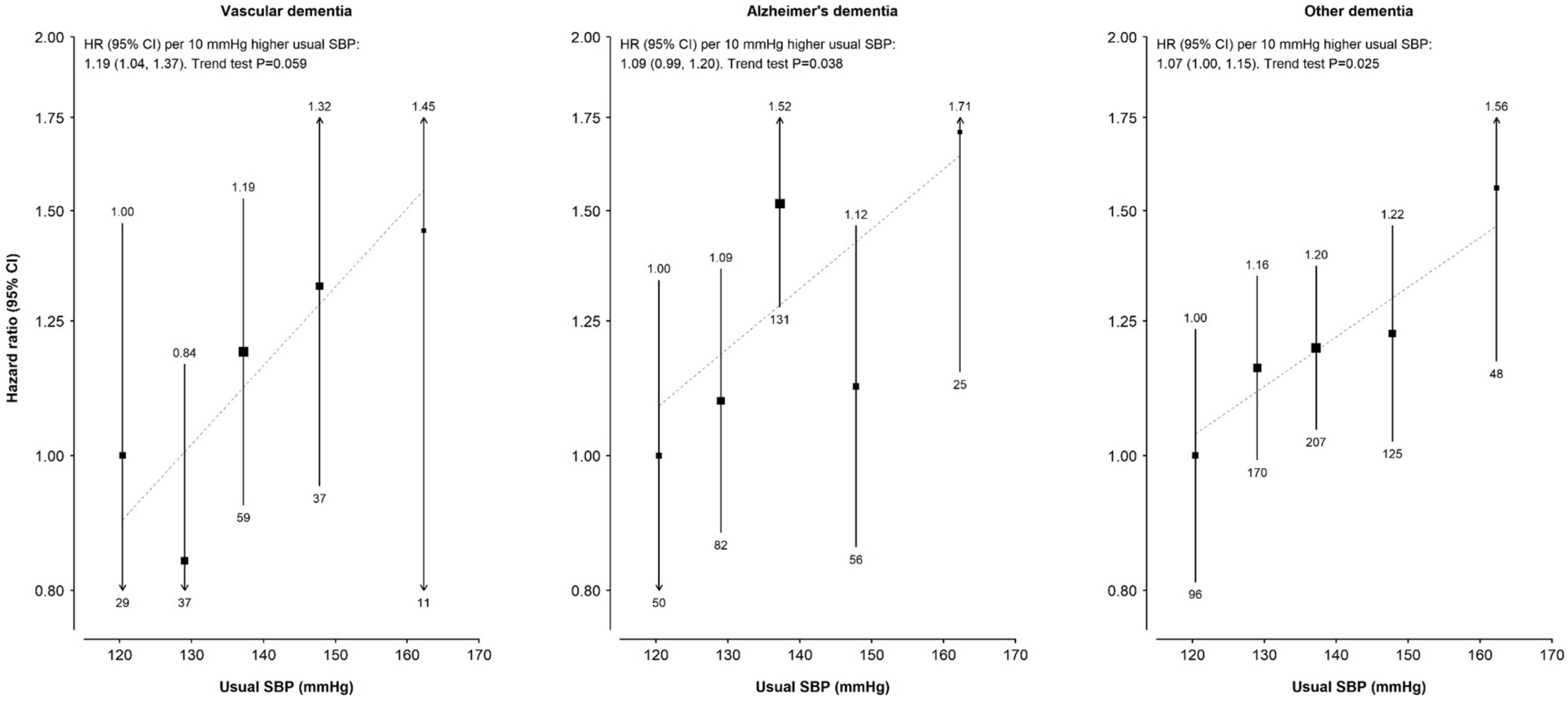

Each square represents hazard ratio (HR) with the area inversely proportional to the variance of the category specific log HR. HRs are plotted on a floating absolute risk scale. Vertical lines indicate 95% CIs. The HRs are shown above each square and numbers of events below. Chi-squared tests were applied to stratum-specific log HRs, using inverse-variance weights to assess evidence of a linear gradient across ordered strata. HRs were stratified by age-at-risk (5-year age groups), sex and region and adjusted for smoking, alcohol consumption, education, physical activity, healthy diet score, and BMI. The regression dilution ratio (RDR=0.50) for estimating usual SBP values was calculated using Rosner's regression method adjusting for region, sex, and 10-year age group. HRs are plotted against the mean usual SBP in each group, estimated by (B-A) x RDR + A, where A is the overall baseline mean SBP, and B is the baseline SBP group mean.

eFigure 7: Association of usual RPG with risk of all-cause dementia and brain atrophy, among participants without a prior history of diabetes at recruitment

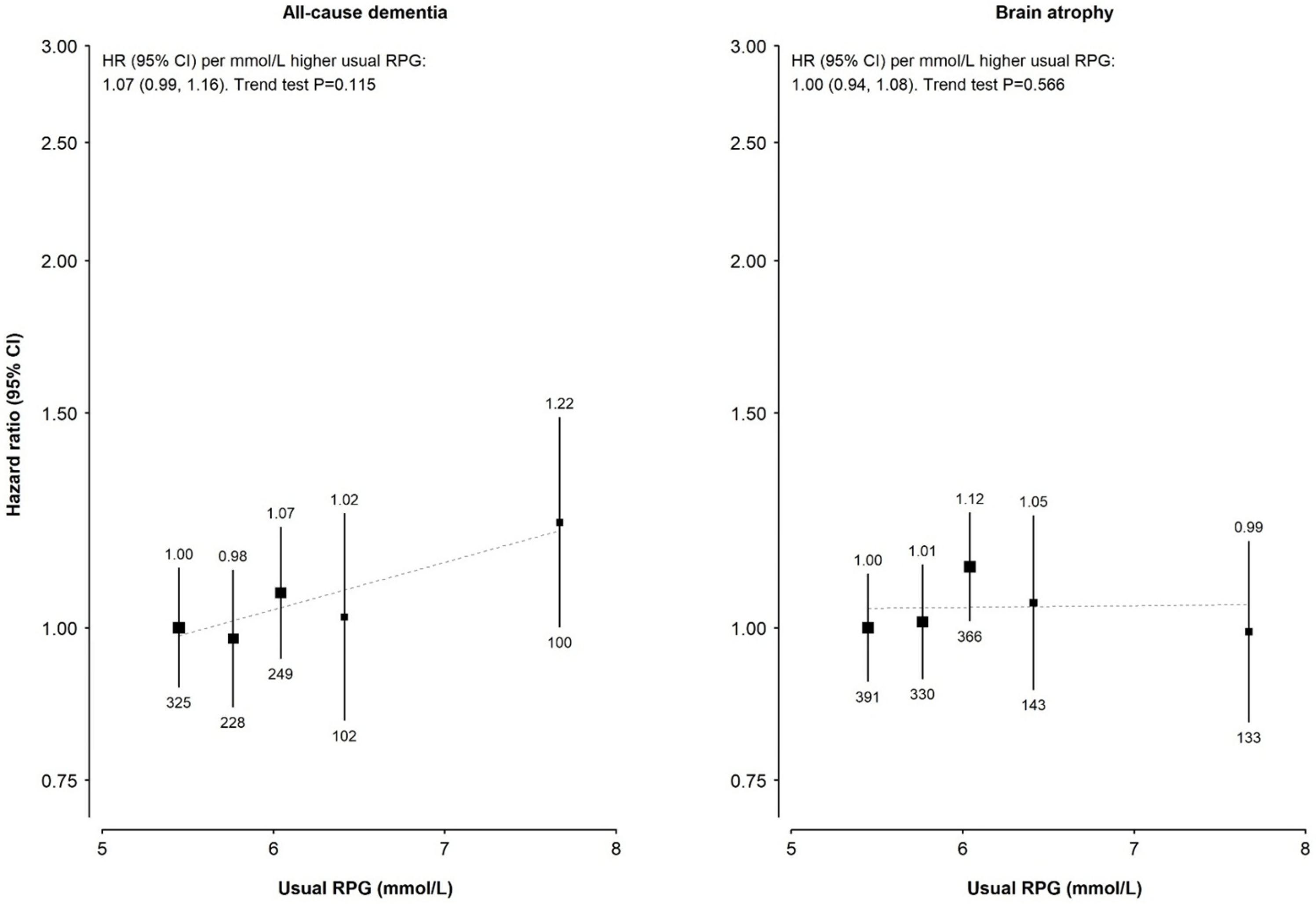

Conventions as eFigure 6. The regression dilution ratio (RDR=0.37) for estimating usual RPG values was calculated using Rosner's regression method adjusting for region, sex, 10-year age group, and hours since last eating. HRs are plotted against the mean usual RPG in each group, estimated by (B-A) x RDR + A, where A is the overall baseline mean RPG, and B is the baseline RPG group mean.

eFigure 8: Association of usual RPG with risk of dementia subtypes, among participants without a prior history of diabetes at recruitment

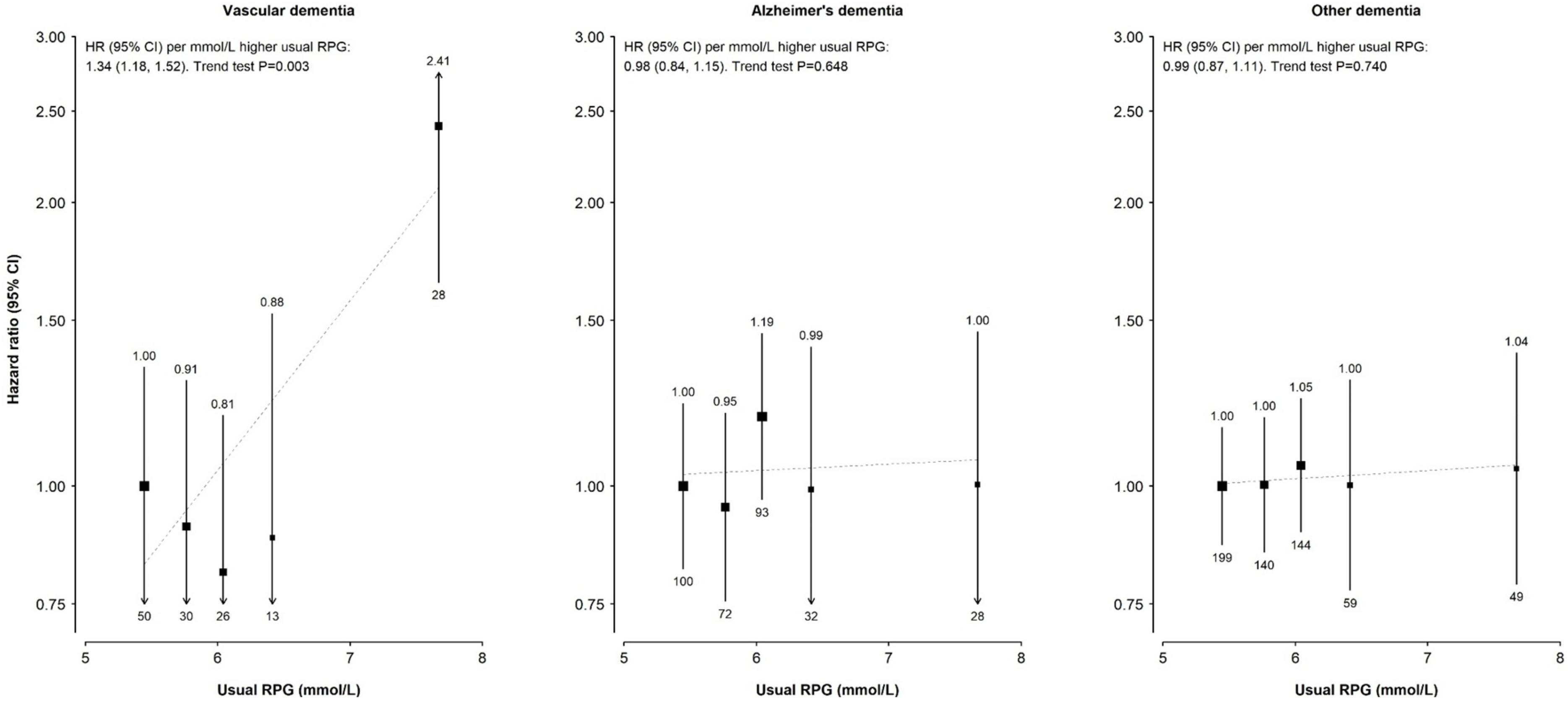

Conventions as eFigure 7.

eFigure 9: Association of hypertension with risk of all-cause dementia and brain atrophy, by baseline characteristics

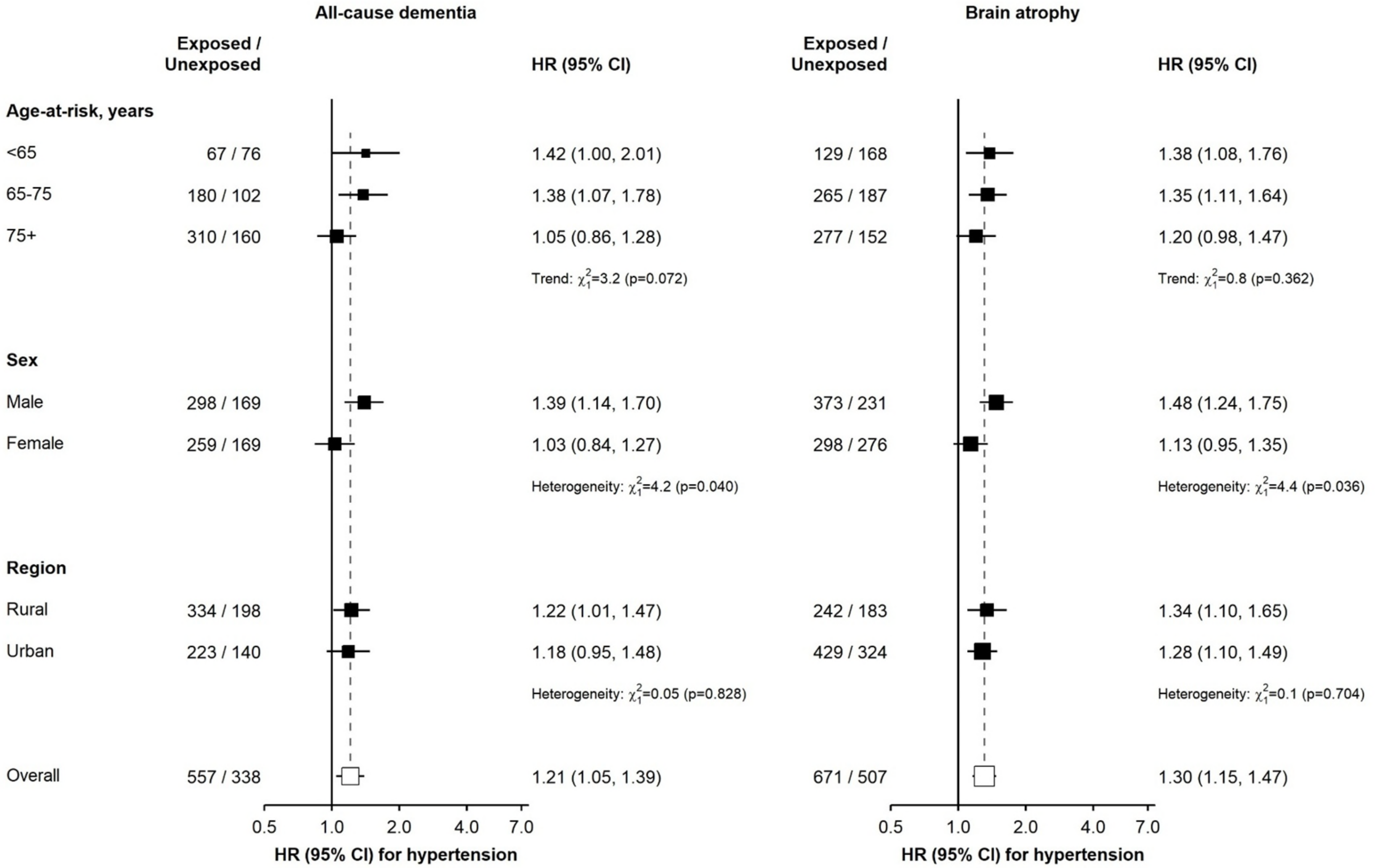

The adjusted hazard ratios (HRs) are shown as squares (scaled proportional to the variance of the log risk) and 95% CIs are shown as horizontal lines. HRs were stratified by age-at-risk (5-year age groups), sex and region and adjusted for smoking, alcohol consumption, education, physical activity, healthy diet score, and BMI. The chi-square and p-values are shown for tests of trend and heterogeneity between subgroups. Exposure is self-reported or screen-detected at recruitment or the first instance of the exposure identified by ICD-10 codes recorded at hospitalisation or in disease registries at least one year prior to incident dementia or brain atrophy.

eFigure 10: Association of diabetes with risk of all-cause dementia and brain atrophy, by baseline characteristics

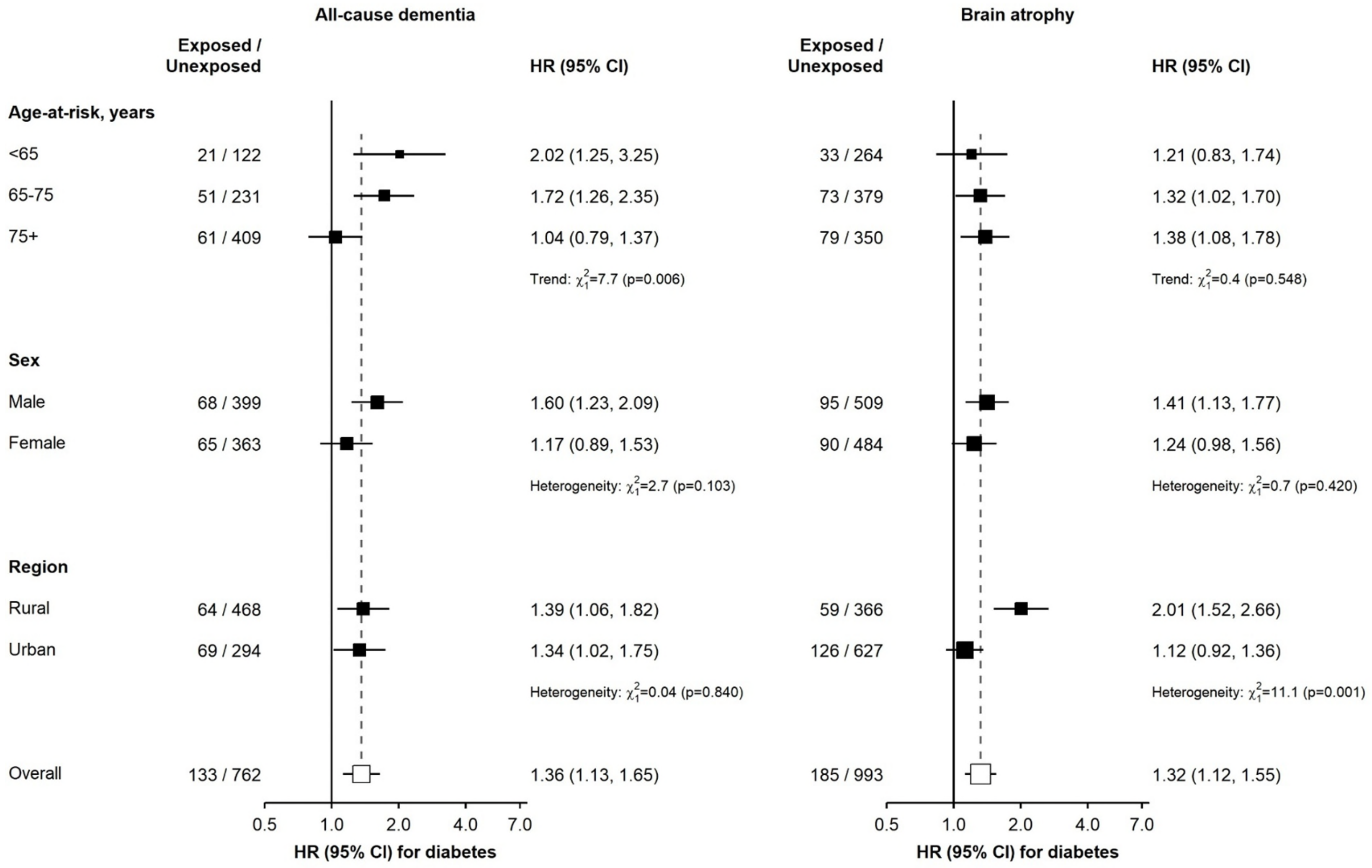

Conventions as eFigure 9.

eFigure 11: Association of IHD with risk of all-cause dementia and brain atrophy, by baseline characteristics

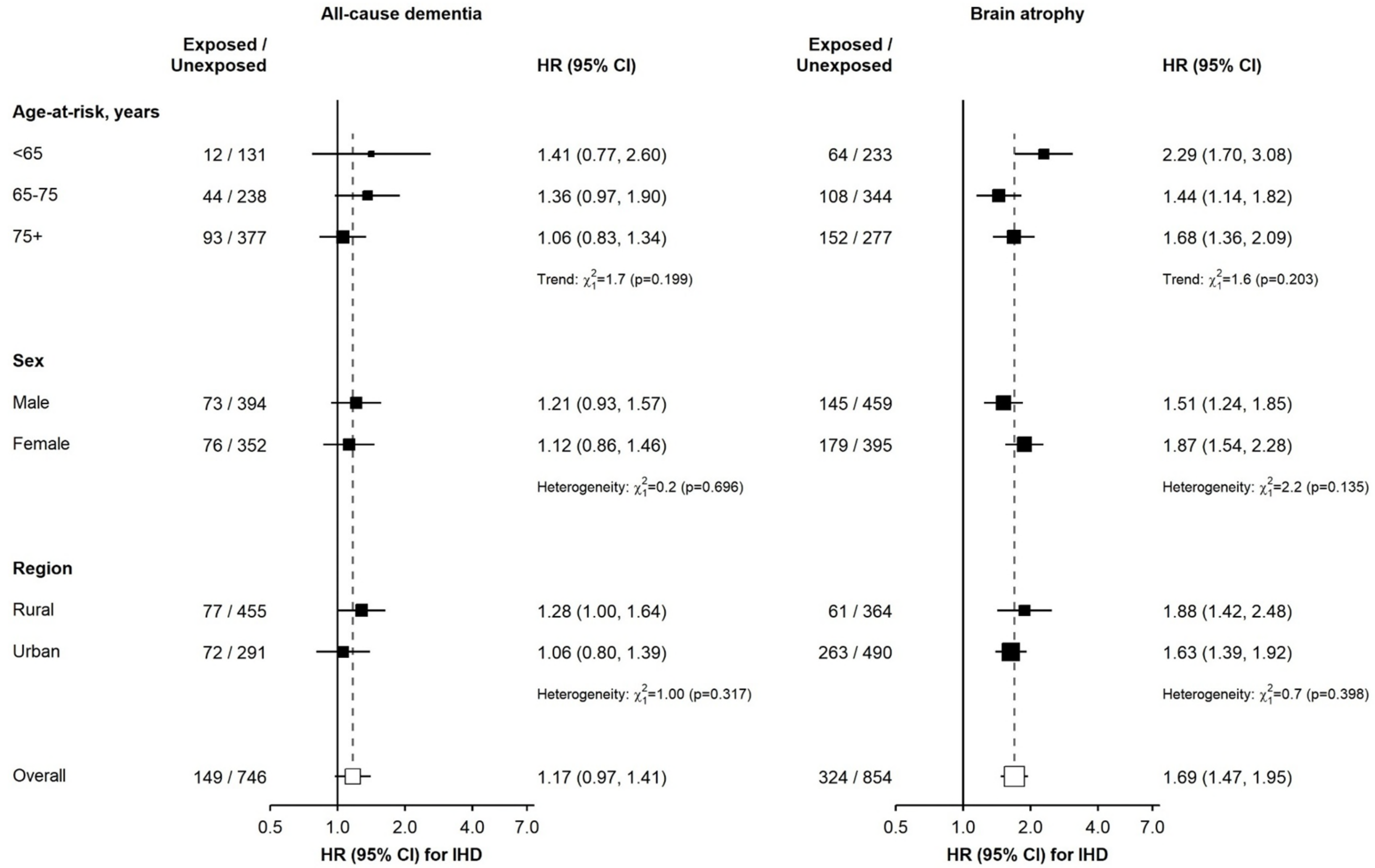

Conventions as eFigure 9. IHD: ischaemic heart disease.

eFigure 12: Association of stroke/TIA with risk of all-cause dementia and brain atrophy, by baseline characteristics

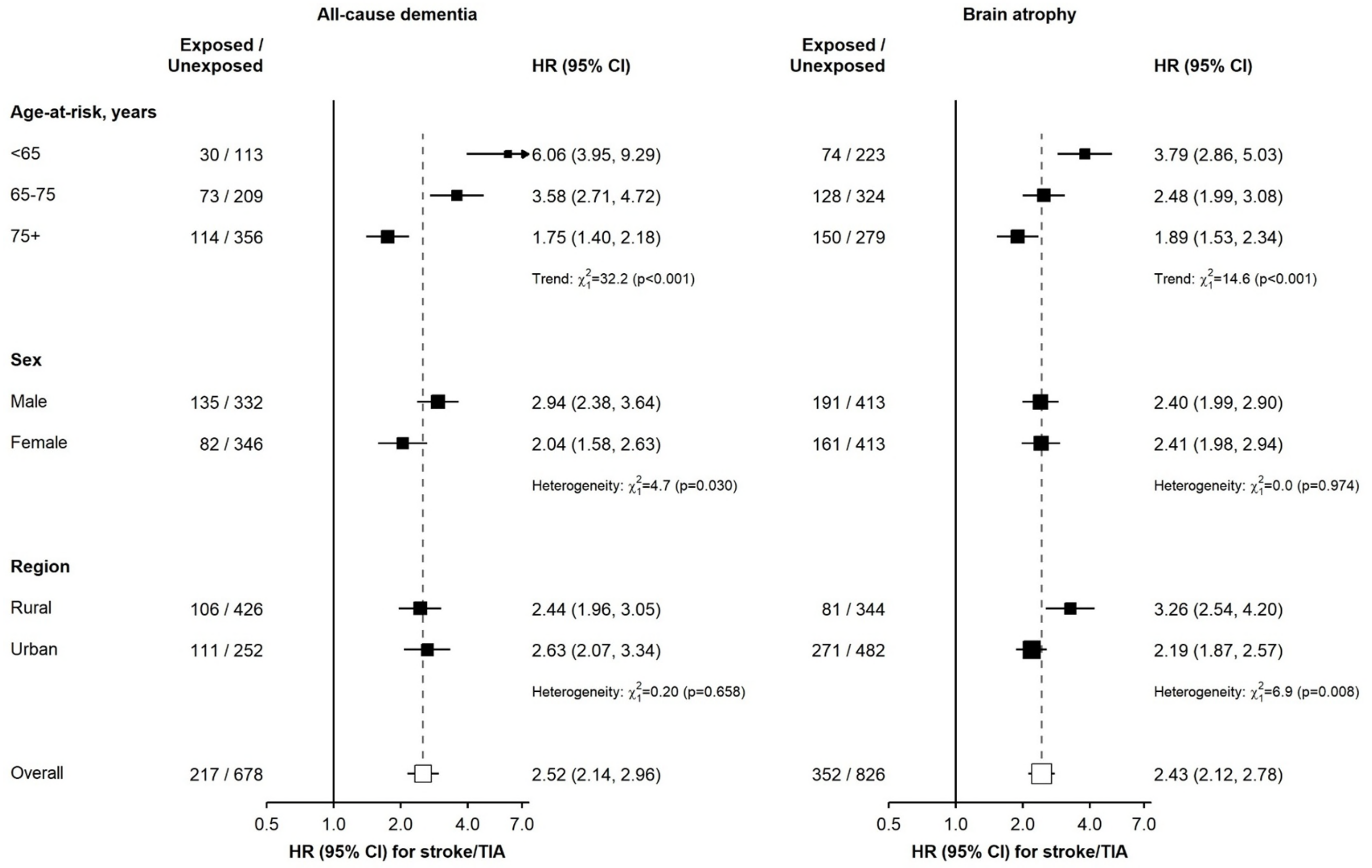

Conventions as eFigure 9. TIA: transient ischaemic attack.

eFigure 13: Associations of markers of prior cardiometabolic health with risk of dementia and brain atrophy at age-at-risk ≥65 years

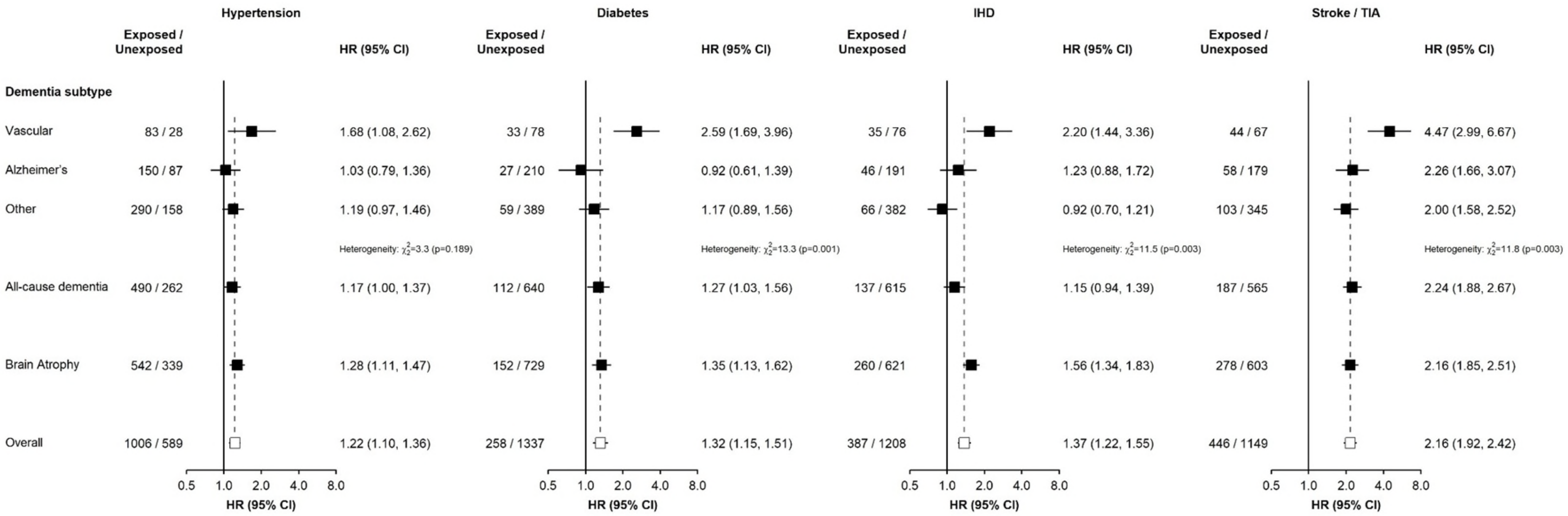

The adjusted hazard ratios (HRs) are shown as squares (not scaled proportional to the variance of the log risk) and 95% CIs are shown as horizontal lines. HRs were stratified by age-at-risk (5-year age groups), sex and region and adjusted for smoking, alcohol consumption, education, physical activity, healthy diet score, and BMI. The chi-square and P-values are shown for heterogeneity between dementia subtypes. Exposure is self-reported or screen-detected at recruitment or the first instance of the exposure identified by ICD-10 codes recorded at hospitalisation or in disease registries at least one year prior to incident dementia or brain atrophy. IHD: ischaemic heart disease; TIA: transient ischaemic attack.

eFigure 14: Associations of markers of cardiometabolic health with risk of dementia and brain atrophy after introducing various time lags between exposure diagnosis and outcomes

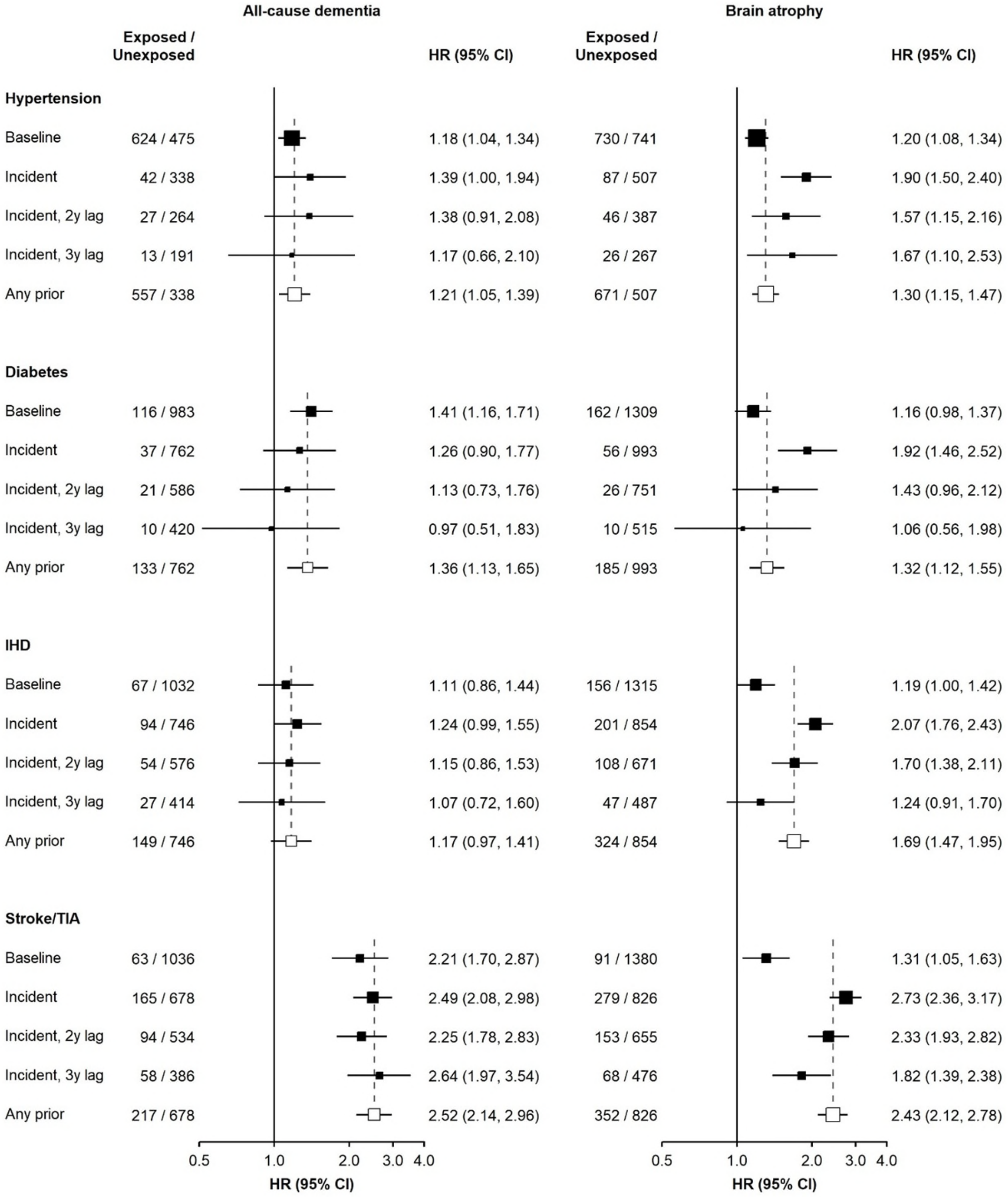

The adjusted hazard ratios (HRs) are shown as squares (scaled proportional to the variance of the log risk) and 95% CIs are shown as horizontal lines. HRs were stratified by age-at-risk (5-year age groups), sex and region and adjusted for smoking, alcohol consumption, education, physical activity, healthy diet score, and BMI. ‘Baseline’ exposure refers to self-reported or screen-detected (for diabetes and hypertension) diagnoses at recruitment. ‘Incident’ exposure refers to first instance of the exposure identified by ICD-10 codes recorded at or during hospitalization or in disease registries preceding incident dementia or brain atrophy by > 1 year (‘incident’), > 2 years, and > 3 years. ‘Any prior’ exposure combines baseline and incident (>1 year) exposure. IHD: ischaemic heart disease; TIA: transient ischaemic attack.

eFigure 15: Associations of markers of prior cardiometabolic health with risk of brain atrophy, excluding participants from Harbin

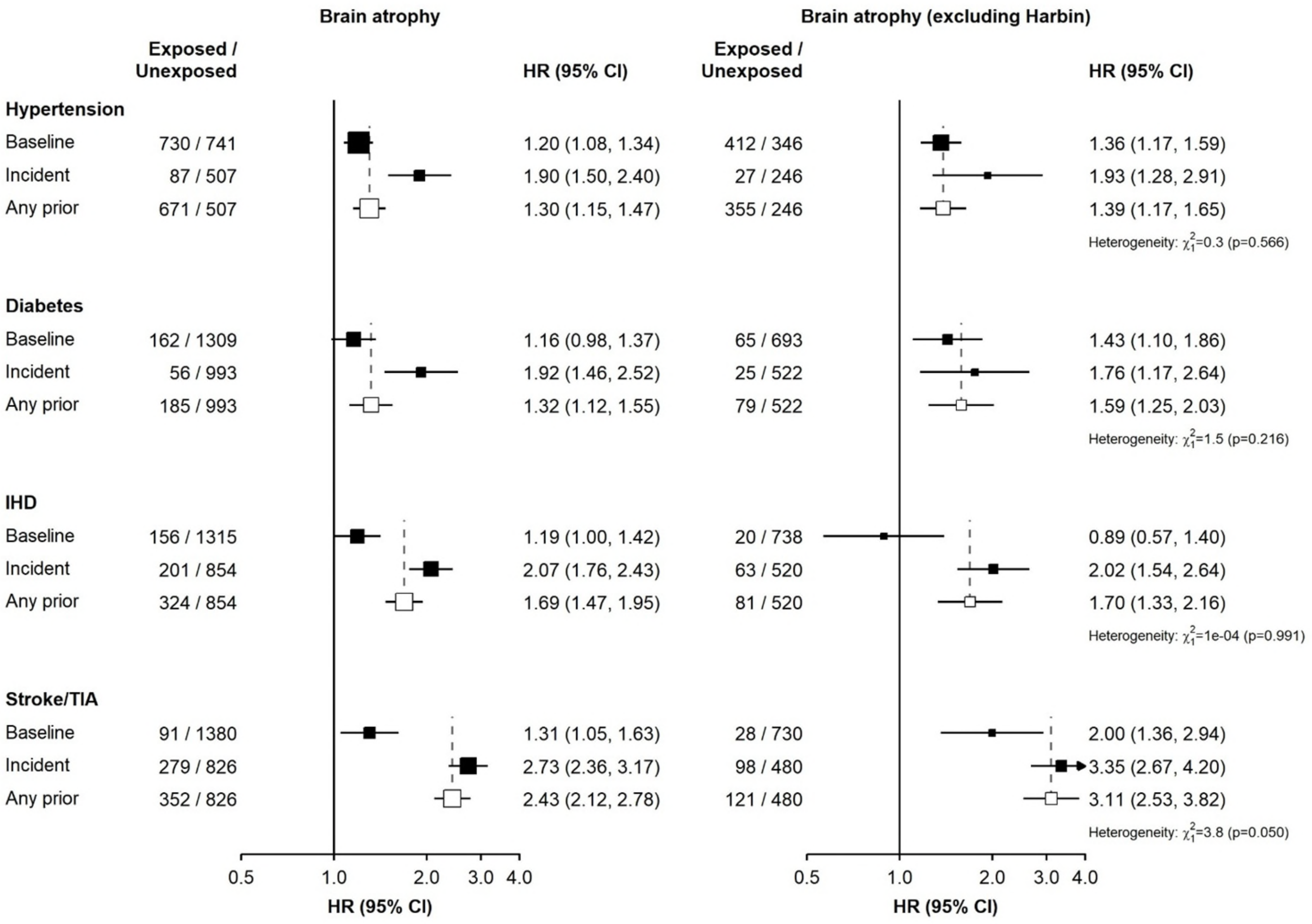

Conventions as eFigure 14. The chi-square and P-values are shown for heterogeneity between any prior exposure including and excluding participants from Harbin.

eFigure 16: Associations of markers of cardiometabolic health at recruitment with risk of all-cause dementia and brain atrophy, by duration of follow-up

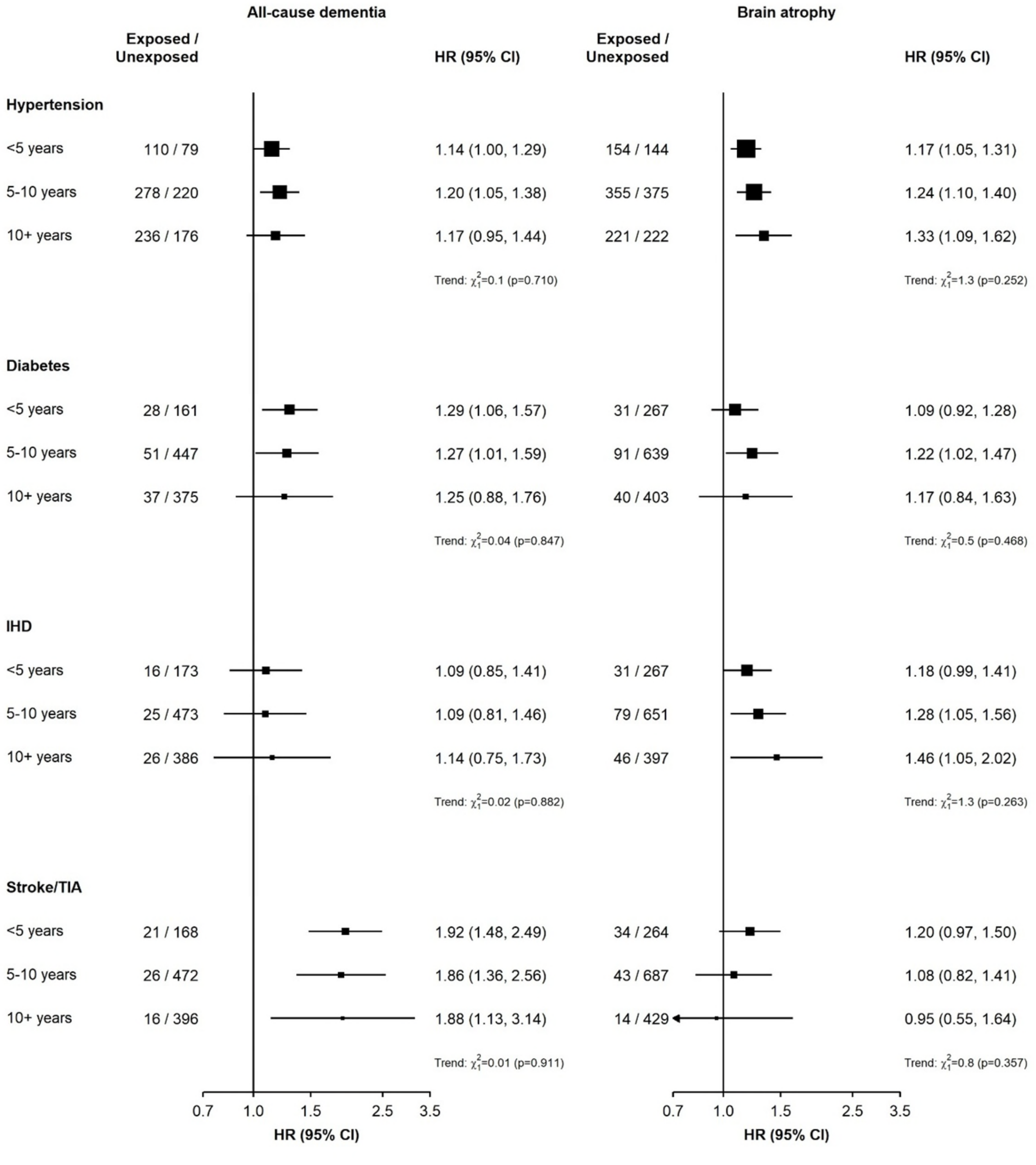

The adjusted hazard ratios (HRs) are shown as squares (scaled proportional to the variance of the log risk) and 95% CIs are shown as horizontal lines. Chi-squared tests were applied to stratum-specific log HRs, using inverse-variance weights to assess evidence of a linear gradient across ordered strata. HRs were stratified by age-at-risk (5-year age groups), sex and region and adjusted for smoking, alcohol consumption, education, physical activity, healthy diet score, and BMI. Exposure is self-reported or screen-detected at recruitment (‘baseline’). Participants who developed the outcome or were otherwise censored before the follow-up period of interest were excluded. IHD: ischaemic heart disease; TIA: transient ischaemic attack.

eFigure 17: Associations of hypertension and diabetes subgroups at recruitment with risk of all-cause dementia and brain atrophy

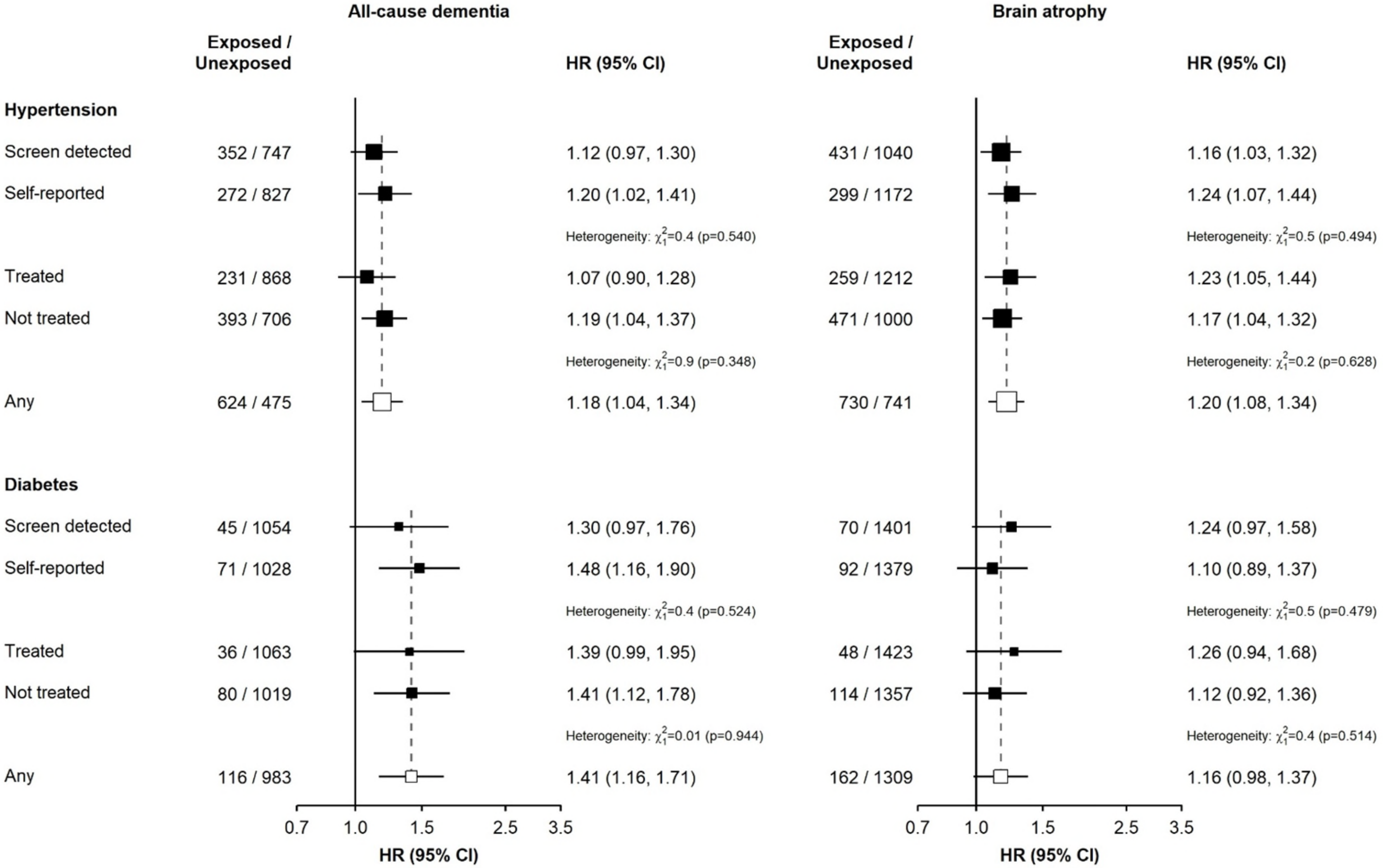

The adjusted hazard ratios (HRs) are shown as squares (scaled proportional to the variance of the log risk) and 95% CIs are shown as horizontal lines. The chi-square and P-values are shown for heterogeneity between detection and treatment subgroups. HRs were stratified by age-at-risk (5-year age groups), sex and region and adjusted for smoking, alcohol consumption, education, physical activity, healthy diet score, and BMI. Exposure is self-reported or screen-detected at recruitment (‘baseline’). The subgroup analyses excludes participants at recruitment with the exposure (hypertension/diabetes) not in the subgroup of interest (self-reported/screening, treated/not treated). Treatment of diabetes is limited to individuals with self-reported diabetes at recruitment.
